# Clinical effect and antiviral mechanism of T-705 in treating severe fever with thrombocytopenia syndrome

**DOI:** 10.1038/s41392-021-00541-3

**Published:** 2021-04-16

**Authors:** Hao Li, Xia-Ming Jiang, Ning Cui, Chun Yuan, Shao-Fei Zhang, Qing-Bin Lu, Zhen-Dong Yang, Qin-Lin Xin, Ya-Bin Song, Xiao-Ai Zhang, Hai-Zhou Liu, Juan Du, Xue-Juan Fan, Lan Yuan, Yi-Mei Yuan, Zhen Wang, Juan Wang, Lan Zhang, Dong-Na Zhang, Zhi-Bo Wang, Ke Dai, Jie-Ying Bai, Zhao-Nian Hao, Hang Fan, Li-Qun Fang, Gengfu Xiao, Yang Yang, Ke Peng, Hong-Quan Wang, Jian-Xiong Li, Lei-Ke Zhang, Wei Liu

**Affiliations:** 1 Beijing Institute of Microbiology and Epidemiology, State Key Laboratory of Pathogen and Biosecurity, Beijing, P. R. China; 2 State Key Laboratory of Virology, Wuhan Institute of Virology, Center for Biosafety Mega-Science, Chinese Academy of Sciences, Wuhan, Hubei P. R. China; 3 The 154 Hospital, People’s Liberation Army, Xinyang, Henan P. R. China; 4 School of Public Health, Peking University, Beijing, P. R. China; 5 Institute of Molecular Medicine, Peking University, Beijing, P. R. China; 6 Tongji Medical College, Huazhong University of Science and Technology, Wuhan, P. R. China; 7 Department of Biostatistics and Emerging Pathogens Institute, University of Florida, Gainesville, FL USA; 8 Department of Cancer, People’s Liberation Army General Hospital, Beijing, P. R. China; 9 Beijing Key Laboratory of Vector Borne and Natural Focus Infectious Diseases, Beijing, P. R. China

**Keywords:** Infection, Microbiology

## Abstract

Severe fever with thrombocytopenia syndrome (SFTS) virus (SFTSV) is an emerging tick-borne virus with high fatality and an expanding endemic. Currently, effective anti-SFTSV intervention remains unavailable. Favipiravir (T-705) was recently reported to show in vitro and in animal model antiviral efficacy against SFTSV. Here, we conducted a single-blind, randomized controlled trial to assess the efficacy and safety of T-705 in treating SFTS (Chinese Clinical Trial Registry website, number ChiCTR1900023350). From May to August 2018, laboratory-confirmed SFTS patients were recruited from a designated hospital and randomly assigned to receive oral T-705 in combination with supportive care or supportive care only. Fatal outcome occurred in 9.5% (7/74) of T-705 treated patients and 18.3% (13/71) of controls (odds ratio, 0.466, 95% CI, 0.174–1.247). Cox regression showed a significant reduction in case fatality rate (CFR) with an adjusted hazard ratio of 0.366 (95% CI, 0.142–0.944). Among the low-viral load subgroup (RT-PCR cycle threshold ≥26), T-705 treatment significantly reduced CFR from 11.5 to 1.6% (*P* = 0.029), while no between-arm difference was observed in the high-viral load subgroup (RT-PCR cycle threshold <26). The T-705-treated group showed shorter viral clearance, lower incidence of hemorrhagic signs, and faster recovery of laboratory abnormities compared with the controls. The in vitro and animal experiments demonstrated that the antiviral efficacies of T-705 were proportionally induced by SFTSV mutation rates, particularly from two transition mutation types. The mutation analyses on T-705-treated serum samples disclosed a partially consistent mutagenesis pattern as those of the in vitro or animal experiments in reducing the SFTSV viral loads, further supporting the anti-SFTSV effect of T-705, especially for the low-viral loads.

## Introduction

Severe fever with thrombocytopenia syndrome (SFTS), an emerging and severe hemorrhagic fever disease caused by a bunyavirus (SFTS virus, SFTSV), was first discovered in China in 2009 and subsequently reported in South Korea and Japan in 2012, with a high case fatality rate (CFR) ranging from 12 to 50%.^1–4^ Increasing number of SFTS cases was soon recorded in these countries, with cumulative numbers of cases by 2018 attaining 11995 in China (data from the National Health Commission of the People’s Republic of China), 866 in South Korea (data from the Korea Centers for Disease Control and Prevention), and 395 in Japan (data from the Japanese National Institute of Infectious Diseases). Two new patients were recently reported in Vietnam,^5^ indicating a further geographical expansion in Asia. Tick-to-human transmission is the primary infection route, with *Haemaphysalis longicornis* tick as the predominant vector of SFTSV.^6,7^ This tick species has very recently been reported spreading widely in several continents of the world, including Asia, Oceania, and North America.^8^ Human-to-human transmission has also been seen in a few family and nosocomial clusters of cases, and the most common risk of the transmission is direct blood exposure.^9,10^ The potential droplet transmission has been posed due to SFTSV RNA was continuously detected in the sputum.^11,12^ The possible sexual transmission has recently been postulated as that SFTSV RNA was detected in semen after the virus had already disappeared from serum.^13^ All these findings raised the possibility of pandemic transmission of SFTSV outside of Asia, and the high risk of human-to-human transmission other than through blood. On the other hand, novel SFTSV-like viruses continued to be detected or isolated.^14–16^ The most prominent example is the Heartland virus, which was first isolated from patients in the USA in 2012 and caused severe febrile disease and even death that resembled the clinical picture of SFTS.^14,17^

Under the current situation of no vaccine available, identification of effective anti-SFTSV drug is critical for treating potential pandemic SFTS or SFTS-like disease transmission on a global level. Current treatment options for SFTS included antiviral therapy and supportive therapy. Ribavirin, which was suggested as a potential anti-SFTSV drug, was reported to be ineffective for improving the disease outcome in a retrospective study,^18^ or with limited efficacy when used early among patients with very low viremia.^4^ Investigation on more effective antiviral therapies became urgent as the epidemics of SFTS continue.

Favipiravir (T-705) is a new anti-influenza drug approved for human use in Japan and is progressing through Phase 3 clinical trials in the USA.^19^ The compound demonstrated strong inhibition activity against a broad spectrum of RNA viruses in vitro or in animal models, including flaviviruses,^20^ arenaviruses,^20–23^ filoviruses,^24,25^ and some members in the family of Bunyaviridae (i.e., Rift Valley fever virus, Sandfly fever virus, and Punta Toro virus).^21,26,27^ Differing from ribavirin that acts by GTP depletion via inhibition of IMP dehydrogenase, T-705 functions as a GTP-competitive inhibitor of the viral polymerase.^28,29^ The main mechanism of action for T-705 has been attributed to be an RNA chain terminator or a lethal mutagen for several RNA viruses in vitro.^30^ Recently, T-705 was shown to attain a higher antiviral efficacy on inhibiting SFTSV replication in vitro and in animal models than ribavirin.^31^ All these findings point to T-705 as a potential antiviral candidate against SFTSV, as well as the above-mentioned RNA viruses. However, a randomized controlled clinical trial to rigorously evaluate the efficacy of T-705 for any of these RNA viruses has yet been lacking in the literature, mostly due to the limited availability of patients to conduct such trials. Moreover, no study has investigated the main mechanism of action for T-705 in treating SFTSV infection in vitro or in vivo.

Here, we assessed the inhibition effect of T-705 on SFTSV infection in vitro and in the animal model and performed deep sequencing to investigate the anti-SFTSV mechanism. Next, we conducted an investigator-initiated, randomized, open-label, single-blind clinical trial to evaluate the effectiveness and safety of T-705 for treating SFTS, and also investigated the mutagenesis pattern of action of T-705 against SFTSV on patient samples.

## Results

### Mechanism of T-705 against SFTSV in vitro

**Fig. 1:**
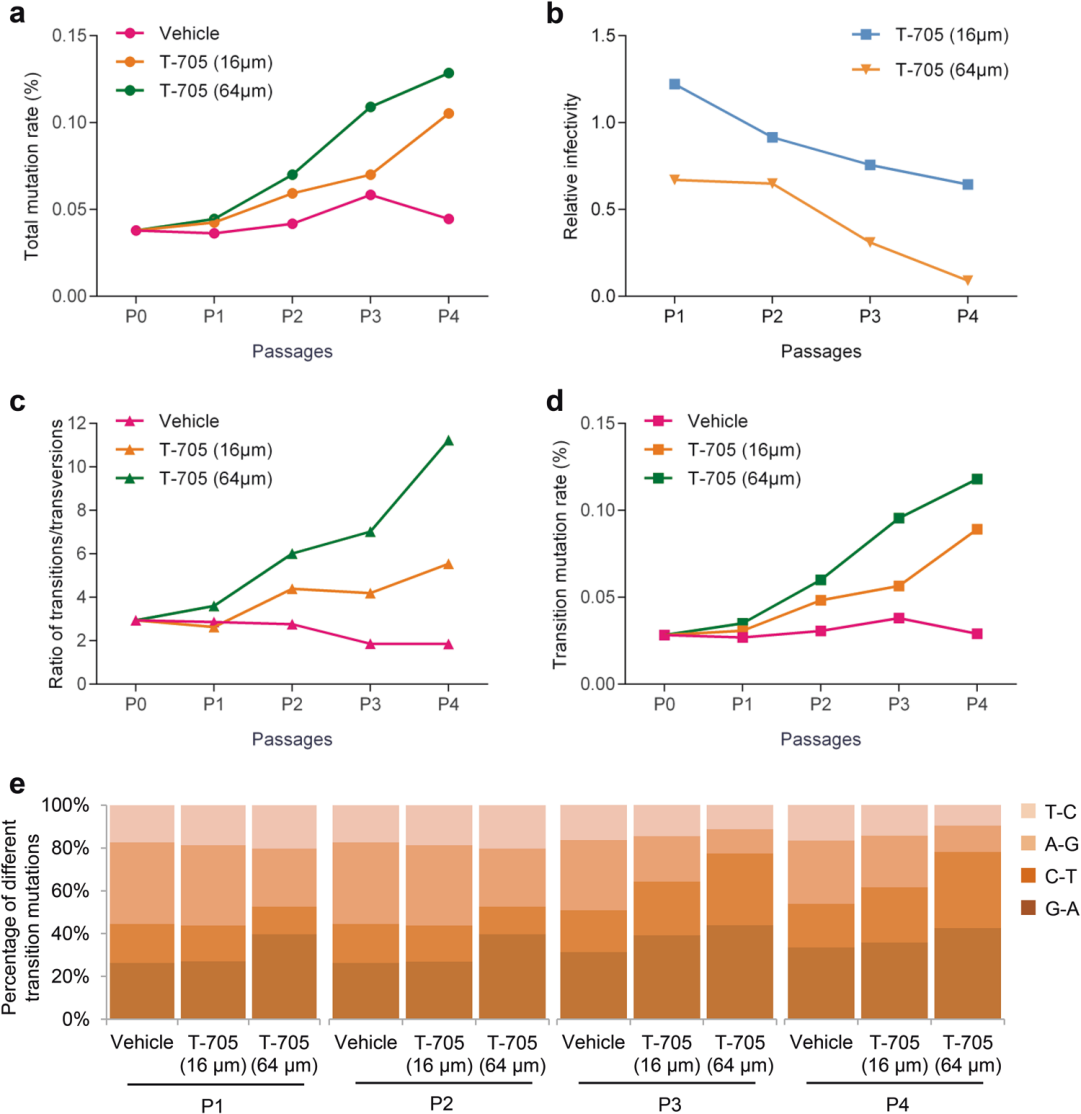
Infectivity and mutation analysis of SFTSV in four passages of cell culture supernatant from cells treated with different concentrations of T-705. SFTSV strain HNXY2017-66 was serial passaged in T-705 or vehicle-treated Vero cells for four passages (P1–P4). The cell culture supernatant was collected for the NGS and mutation analysis of the SFTSV genome. Total mutation rates (**a**), ratios of transitions/transversions (**c**), transition mutation rates (**d**), and percentages of different transition mutations (**e**) were calculated. Viral titer and viral genome copy number in the supernatant were also measured, and relative viral infectivity (viral titer/viral genome copy number) is shown (**b**)

Previous studies have demonstrated that T-705 could inhibit the replication of several viruses of the Bunyavirales in cell culture;^21,26,31^ however, the antiviral mechanism has been investigated in only Rift Valley fever virus (RVFV), a mosquito-borne bunyavirus.^32^ We examined the association between virus extinction and SFTSV mutagenesis that was induced by T-705 in cell culture under different drug concentrations, various virus passages, and different inoculation doses. SFTSV was propagated in Vero cells in the absence or presence of different concentrations of T-705 for four serial passages (P1 to P4). The virus stock solution (designate as P0), and the supernatants and cells harvested from P1 to P4, were separately used for genomic RNA extraction and then subjected to next-generation sequencing (NGS), to determine mutation rates of genomic RNA (Supplementary Fig. S1). For SFTSV genome RNAs extracted from the supernatants, the total mutation rates increased gradually as a result of T-705 treatment, compared to DMSO (vehicle)-treated group (Fig. 1a). Moreover, the total mutation rates increased from P0 to P4, which might be attributed to the accumulation of mutations. T-705 treatment with the concentration of 16 μM showed no apparent anti-SFTSV effect at P1 and P2, and only produced a slight reduction in viral infectivity capacity at P3 and P4, while with the concentration of 64 μM, T-705 treatment showed a remarkable reduction in viral infectivity capacity during the consecutive passages (Fig. 1b). A remarkable increase of the transitions/transversions ratio was observed after treatment with T-705, especially with higher values seen upon the concentration of 64 μM (Fig. 1c), thus revealing that transition mutations (Fig. 1d), instead of transversion mutations, were preferentially induced by T-705. Among four types of transition mutations, the increases of G → A and C → T transitions accounted for the majority (Fig. 1e). For SFTSV genome RNAs extracted from the cells, we found T-705 treatment produced an increase in viral mutation rates and a reduction of intracellular viral RNA levels (Supplementary Fig. S2). Similar to the supernatants, the mutagenesis pattern featured by high transitions/transversions ratios was also observed in the cells after the T-705 treatment (Supplementary Fig. S2). Taken together, these findings suggested a correlation between the loss of viral infectivity and mutagenesis consequence, both of which were affected by the concentration and action time of T-705.

By examining single-nucleotide variants (SNVs) across viral genomes in cell cultures and supernatants, we found SNVs were widely distributed in L, M, and S segments, and enlarged as the concentration of T-705 increased (Supplementary Fig. S3). Apparently higher frequencies of SNVs were observed in L and M segments other than S segment in the cell culture in P4; however, the different distribution of SNVs in the three segments was not seen in the supernatant in P4 (Supplementary Fig. S3), suggesting that the virus with high SNVs in L and M segments could not be packaged or released into the supernatant. This finding revealed that T-705 had a more significant mutagenic effect on these two segments, which might mainly contribute to the viral lethal.

**Fig. 2:**
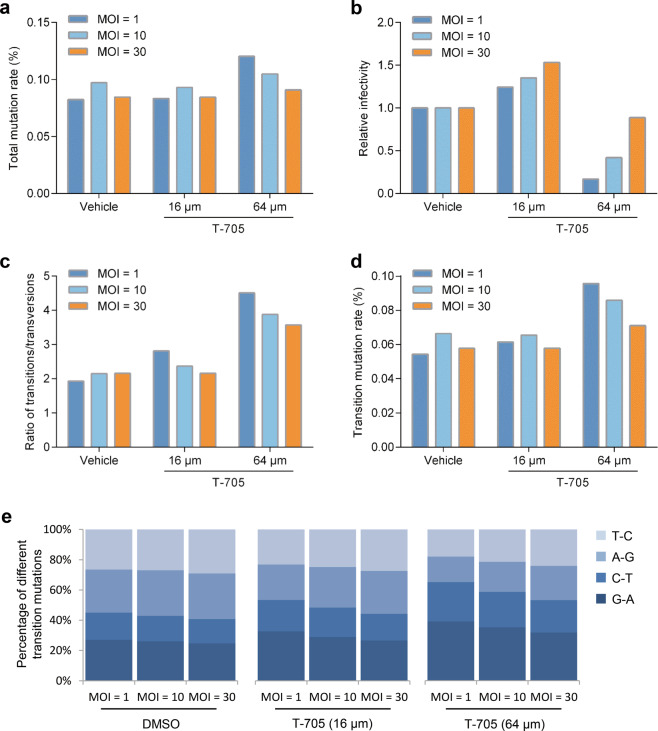
Infectivity and mutation analysis of SFTSV in cell culture supernatant from cells infected with different MOIs of SFTSV. Vero cells were infected with SFTSV strain HNXY2017-66 at MOIs of 1, 10, and 30, and treated with vehicle or different concentrations of T-705. At 24 h post infection, the cell culture supernatant was collected. SFTSV genome in the supernatant was extracted and subjected to NGS analysis. Total mutation rates (**a**), ratios of transitions/transversions (**c**), transition mutation rates (**d**), and percentages of different transition mutations (**e**) were calculated. Viral titer and viral genome copy number in the supernatant were also measured, and relative viral infectivity (Viral titer/viral genome copy number) is shown (**b**)

We further investigated whether the different multiplicity of infections (MOIs) of SFTSV affected the antiviral activity of T-705 in vitro (Supplementary Fig. S4). T-705 treatment with the concentration of 64 μM resulted in a reduction of viral infectivity capacity and an increase of total mutation rates at MOIs of 1 and 10, which was not observed upon the concentration of 16 μM (Fig. 2a, b). When cells were infected with SFTSV at an MOI of 30, T-705 treatment (64 μM) did not have a significant effect on the infective progeny production and virus mutations, implying that T-705 may be less efficient against SFTSV with high viral loads (Fig. 2a, b). Examination of mutation bias revealed that T-705 treatment preferentially induced G → A and C → T transition mutations (Fig. 2d, e), with the consistent increase of transitions/transversion ratios (Fig. 2c). Moreover, similar results were also observed in intracellular viral RNA after the treatment of T-705 (Supplementary Fig. S5). These findings indicated that the mutagenesis patterns induced by T-705 were not affected by viral loads.

### Mechanism of T-705 against SFTSV in a mouse model

**Fig. 3:**
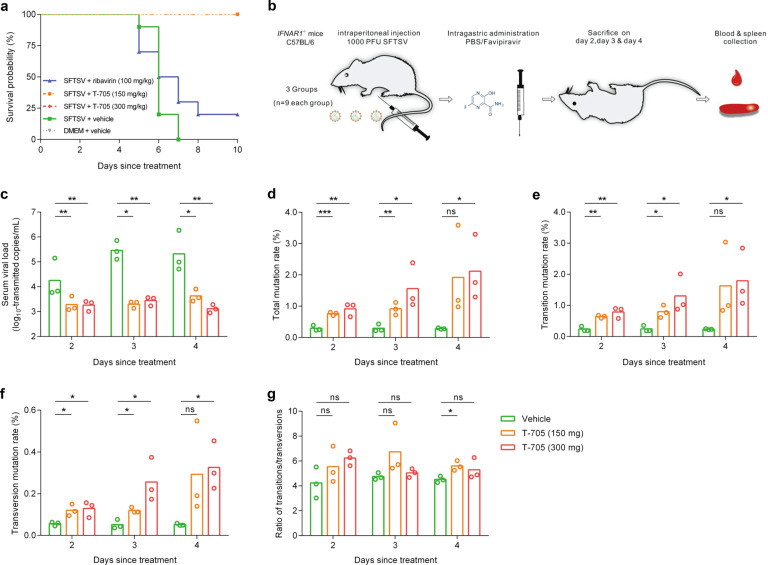
The NGS and mutation analysis of SFTSV genome in serum samples collected from IFNAR^−/−^ C57BL/6 mouse treated with or without T-705. **a** Kaplan–Meier curves for the T-705 treatment effect on the probability of survival in IFNAR^−*/*−^ C57BL/6 mouse. IFNAR^−*/−*^ C57BL mice were divided into five groups: SFTSV + vehicle group (five female and five male mice), SFTSV + T-705 group I (300 mg/kg/d, five female and five male mice), SFTSV + T-705 group II (150 mg/kg/d, five female and five male mice), SFTSV + ribavirin (100 mg/kg/d, five female and five male mice), and DMEM + T-705 group (300 mg/kg/d, three female and three male mice). **b** Flowchart of next-generation sequencing (NGS) of SFTSV genome in spleen and serum samples collected from IFNAR^−*/*−^ C57BL/6 mouse. Three IFNAR^−/−^ C57BL/6 mice from each group were sacrificed on days 2, 3, and 4 post infection, respectively. Serum and spleen samples were collected, and RNA was extracted for NGS analysis. Copy number of SFTSV genome in serum (**c**), the total mutation rates (**d**), transition mutation rates (**e**), transversion mutation rates (**f**), and ratios of transitions/transversions (**g**) were also calculated

We also measured the effect of T-705 on SFTSV replication in IFNAR^*−/−*^ C57BL/6 mouse, a lethal animal model for SFTSV infection.^33^ The IFNAR^*−/−*^ C57BL/6 mice were infected with SFTSV, and then treated with either DMSO (vehicle) or 100 mg/kg/day of ribavirin or 150 mg/kg/day of T-705 or 300 mg/kg/day of T-705 (Supplementary Materials and Methods). We found the survival rates of mice were significantly increased after being treated with T-705 compared with those treated with DMSO or ribavirin (all *P* < 0.05, log-rank test; Fig. 3a). The mutagenesis mechanism of T-705 on RNA viruses has rarely been investigated in vivo.^24,34^ We then investigated the mechanism of T-705 against SFTSV in IFNAR^−*/−*^ C57BL/6 mouse model (Fig. 3b). Significantly reduced viral loads of SFTSV were observed in the serum samples of T-705-treated mice at days 2, 3, 4 post infection (p.i.) (Fig. 3c). As shown in Fig. 3d, simultaneously enhanced virus mutation rates in serum samples were observed in T-705-treated mice than in vehicle-treated mice, except for the treatment of 150 mg/kg/day at day 4 p.i. The total mutation rates increased with the duration of treatment. T-705 treatment of 300 mg/kg/day showed high accumulation of mutation rates than that of 150 mg/kg/day, although no statistical difference was observed (Fig. 3d). The analysis of mutagenesis patterns revealed that the T-705 treatment produced both transition and transversion mutations (Fig. 3e, f), and the ratio of transitions/transversions did not show a significant change (Fig. 3g). No predominant pattern was observed among transition mutations or transversion mutations (Supplementary Fig. S6). We also detected the effect of T-705 on SFTSV RNAs extracted from spleen samples and found significant decreases in viral loads and increases in mutation rates as a result of T-705 treatment (Supplementary Fig. S7). As seen in serum samples, transition and transversion mutations were both significantly induced by T-705 treatment (Supplementary Fig. S7). Although the mutagenesis mechanism of T-705 in treating SFTSV infection in mouse model differed from that in vitro which mainly induced transition mutations, these results highlight the potential of T-705 as an effective agent for inhibition SFTSV replication in vivo through inducing viral lethal mutagenesis.

### Clinical trial participants

**Fig. 4:**
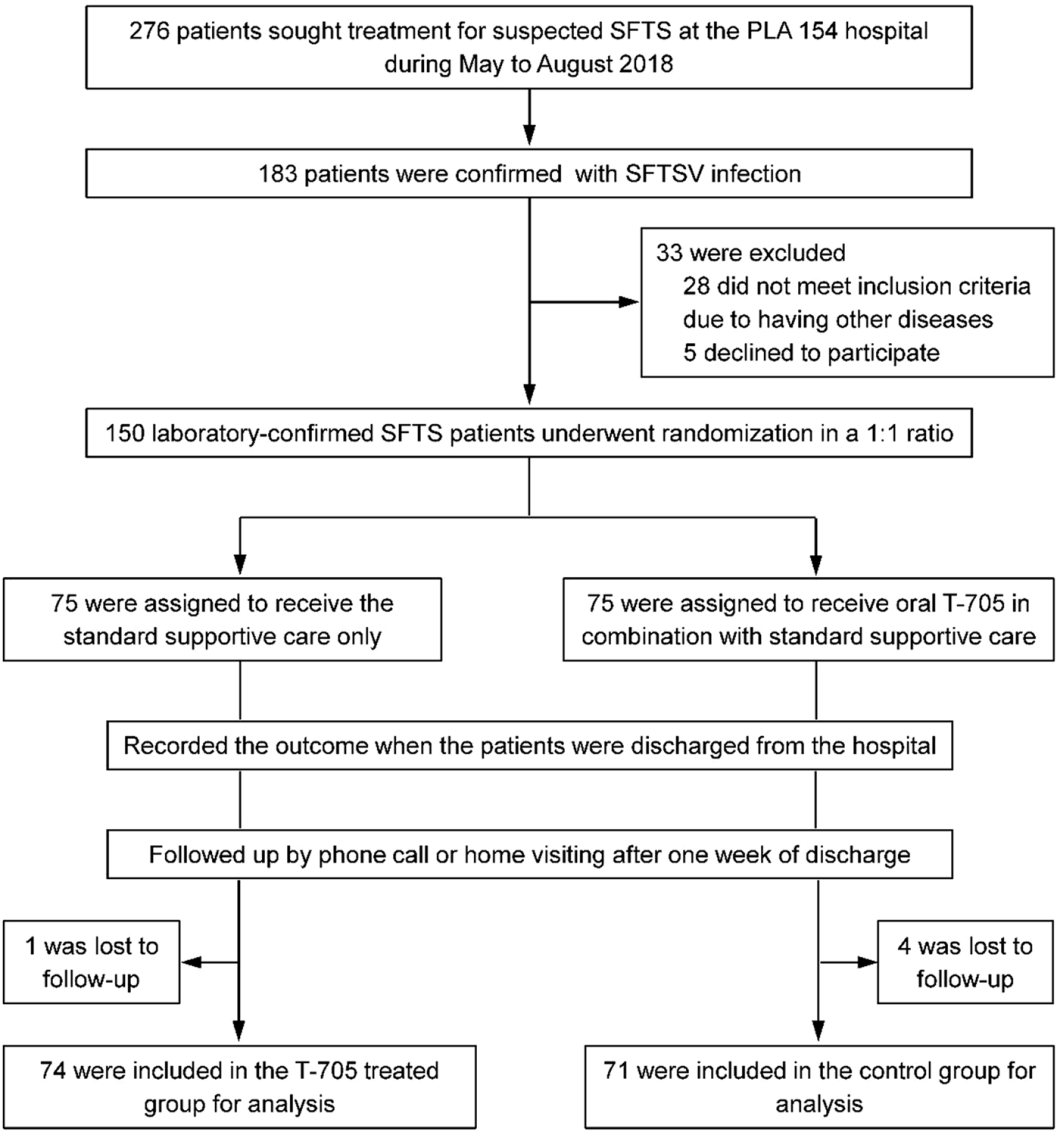
Enrollment, randomization, and follow-up of the study patients. We screened 276 SFTS suspected patients and identified 183 with laboratory-confirmed SFTSV infection. Thirty-three patients were excluded, and 150 were enrolled and randomly assigned to two groups (75 patients in each group). Five patients were lost to follow-up, and eventually 145 were included for analysis

To examine the efficacy and safety of T-705 in treating SFTS, we conducted an investigator-initiated randomized controlled trial in the designated hospital for SFTS treatment in the area of greatest endemicity in China. Patients with laboratory-confirmed SFTS who met the eligibility criteria were recruited into the study. A total of 150 patients (of 276 screened) were enrolled and randomly assigned to two groups (75 patients in each group). Five patients were lost to the follow-up, and eventually, 145 (74 in the T-705-treated group and 71 in the control group) were included for final analysis (Fig. 4). The mean (±SD) age (64.7 ± 12.1 vs. 62.4 ± 12.4 years), gender proportion (female, 70.3% vs. 64.8%), and mean (±SD) days from symptom onset to hospital admission (5.1 ± 1.6 vs. 4.9 ± 1.7) were comparable between the two groups (Table 1). The mean (±SD) RT-PCR cycle-threshold (CT) value examined at the patients’ admission between the T-705-treated group (30.0 ± 3.9) and the control group (30.6 ± 4.5) was similar (*P* = 0.455; Supplementary Fig S8). Demographic data, vital signs, and clinical features, and laboratory test results, were well balanced between the two groups (Table 1 and Supplementary Table S1). The measures that were provided as the standard supportive care included supplement of electrolytes and dextrose, antipyretics, hepatoprotective, a supplement of multivitamins, recombinant human granulocyte colony-stimulating factor, immunopotentiating agents, antiemetics, antibiotics, and plasma transfusion. Each of these measures was administered with comparable frequencies between the two groups (Supplementary Table S2). Overall, the two groups had comparable clinical and laboratory characteristics and viral loads before the intervention, as well as similar supportive treatment measures during hospitalization.

**Table 1:** Baseline demographic characteristics, vital signs, and clinical features of the SFTS patients

Characteristic	T-705-treated group (*n* = 74)	Control group (*n* = 71)	*P* value
Age (yr)	64.7 ± 12.1	62.4 ± 12.4	0.256
Age ≥60 yr, no. (%)	52 (70.3)	46 (64.8)	0.481
Female sex, no. (%)	44 (59.5)	44 (62.0)	0.757
History of tick bites, no. (%)^*^	8 (10.8)	9 (12.7)	0.727
Days from symptom onset to admission	5.1 ± 1.6	4.9 ± 1.7	0.478
*Vital signs*			
Body temperature, °C	38.2 ± 0.9	38.0 ± 1.0	0.086
Pulse, beats/min	85.4 ± 9.8	84.5 ± 8.6	0.552
Respiratory rate, breaths/min	18.7 ± 1.1	18.5 ± 0.9	0.124
Systolic blood pressure, mm Hg	116.0 ± 16.7	113.5 ± 17.5	0.393
Diastolic blood pressure, mm Hg	73.6 ± 12.0	72.0 ± 10.7	0.385
*Clinical features, no. (%)*			
Fever	71 (95.9)	69 (97.2)	1.000
Feeble	71 (95.9)	71 (100)	0.245
Dizziness	13 (17.6)	18 (25.4)	0.253
Headache	4 (5.7)	9 (12.7)	0.126
Chills	10 (13.5)	17 (23.9)	0.107
Myalgias	50 (67.6)	52 (73.2)	0.455
Arthralgia	2 (2.7)	1 (1.4)	1.000
Lymphadenopathy	39 (52.7)	44 (62.0)	0.259
Anorexia	63 (85.1)	65 (91.5)	0.230
Nausea	59 (79.7)	56 (78.9)	0.899
Vomiting	34 (45.9)	27 (38.0)	0.334
Abdominal pain	1 (1.4)	1 (1.4)	1.000
Diarrhea	12 (16.2)	16 (22.5)	0.335
Cough	19 (25.7)	21 (29.6)	0.599
Sputum	13 (17.6)	13 (18.3)	0.907
Pulmonary infection	4 (5.4)	9 (12.7)	0.153
Bronchitis	2 (2.7)	4 (5.6)	0.435
Dyspnea	1 (1.4)	0	1.000
Ophthalmorrhagia	2 (2.7)	0	0.497
Gingival bleeding	1 (1.4)	2 (2.8)	0.615
Ecchymosis	1 (1.4)	3 (4.2)	0.360
Melena	1 (1.4)	1 (1.4)	1.000
Dysphoria	1 (1.4)	1 (1.4)	1.000
Convulsion	0	4 (5.6)	0.055
Confusion	4 (5.4)	2 (2.8)	0.681
Lethargy	5 (6.8)	4 (5.6)	1.000

^*^History of tick bites was self-reported

### Mortality rates in SFTS patients with T-705 treatment

**Fig. 5:**
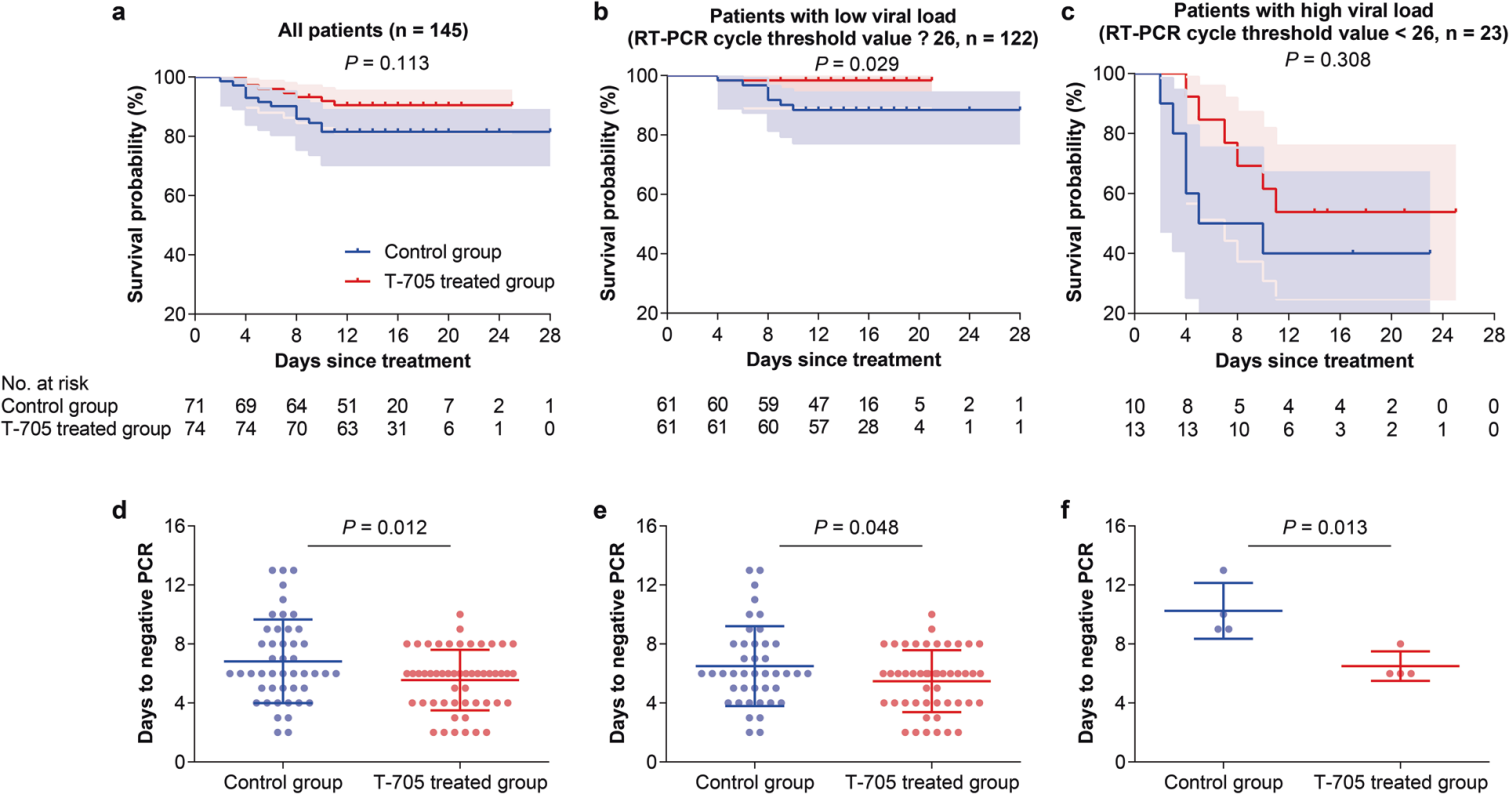
Effect of T-705 treatment on survival time and viral clearance. **a**–**c** Kaplan-Meier curves for T-705 treatment effect on the probability of survival. Kaplan–Meier survival curves with 95% confidence bands are shown by treatment arm for all 145 patients (**a**), for 122 patients (**b**) with low-baseline viral loads (RT-PCR cycle-threshold value ≥26), and for 23 patients (**c**) with high-baseline viral loads (RT-PCR cycle-threshold value <26). **d**–**f** The days to viral clearance of SFTSV RNA. The mean (standard deviation) days to viral clearance are shown for all patients (**d**), for patients (**e**) with low-baseline viral loads, and for patients (**f**) with high-baseline viral loads

Fatal outcome (defined as all-cause death), as the primary outcome, occurred in 7 of the 74 T-705-treated patients (9.5%, 95% CI, 2.8–16.1%) and 13 of the 71 control patients (18.3%, 95% CI, 9.3–27.3%), giving an odds ratio of 0.47 (95% CI, 0.17–1.25) in case fatality rate (CFR). The survival curves showed no inter-group difference (*P* = 0.113, log-rank test; Fig. 5a). However, the Cox regression showed a significant reduction in CFR with a hazard ratio of 0.37 (95% CI, 0.14–0.94; *P* = 0.038), after adjustment for age, gender, and delay from symptom onset to hospital admission. Stratified analysis was further performed to evaluate the treatment efficacy. We found that the treatment efficacy differed by the delay from symptom onset to hospital admission and by baseline viral load. Among the patients with a delay <6 days, T-705 treatment decreased the CFR from 19.6 (10 of 51) to 4.2% (2 of 48) (*P* = 0.021, log-rank test; Supplementary Fig. S9), whereas no treatment effect was found for the patients with a delay ≥6 days (*P* = 0.810; Supplementary Fig. S9). At enrollment, 82.4% (61 of 74) of the T-705 group and (61 of 71) 85.9% of the control group had relatively low baseline levels of viral load (RT-PCR CT ≥ 26) (*P* = 0.566; Supplementary Fig. S8). Among the low viral load subgroup, T-705 treatment was associated with a significantly reduced CFR, 1.6% (1 of 61), compared to 11.5% (7 of 61) in the control group (*P* = 0.029; Fig. 5b). In contrast, no between-arm difference was observed in the patients with high-baseline viral load (RT-PCR CT < 26), with the CFR moderately reduced from 60.0% (6 of 10) in the control group to 46.2% (6 of 13) in the T-705-treated group (*P* = 0.308; Fig. 5c). Age and gender, on the other hand, showed no significant effect in influencing the treatment effect (all *P* > 0.05; Supplementary Fig. S9).

### Viral clearance in SFTS patients with T-705 treatment

In this clinical trial, the time needed for viral clearance (defined as the time to a first negative RT-PCR assay) and dynamic changes of viral load during hospitalization were analyzed as the secondary outcomes. The mean (±SD) days to viral clearance was 5.6 ± 2.1 in the T-705-treated group, significantly shorter than that (6.8 ± 2.8) in the control group (*P* = 0.012; Fig. 5d). Consistent results were observed in the patients with either low baseline viral loads (5.5 ± 2.1 vs. 6.5 ± 2.7 days, *P* = 0.048; Fig. 5f) or high-baseline viral loads (6.5 ± 1.0 vs. 10.3 ± 1.9 days, *P* = 0.013; Fig. 5f). Moreover, a more rapid decrease of viral load was associated with T-705 treatment when all the patients were considered by using the generalized estimating equation (GEE) model (*P* = 0.024; Supplementary Fig. S10), which was also observed in the patients with low-baseline viral loads (*P* = 0.024; Supplementary Fig. S10), however, the patients with high-baseline viral loads had too little data to assess the association.

### Clinical improvement in SFTS patients with T-705 treatment

**Fig. 6:**
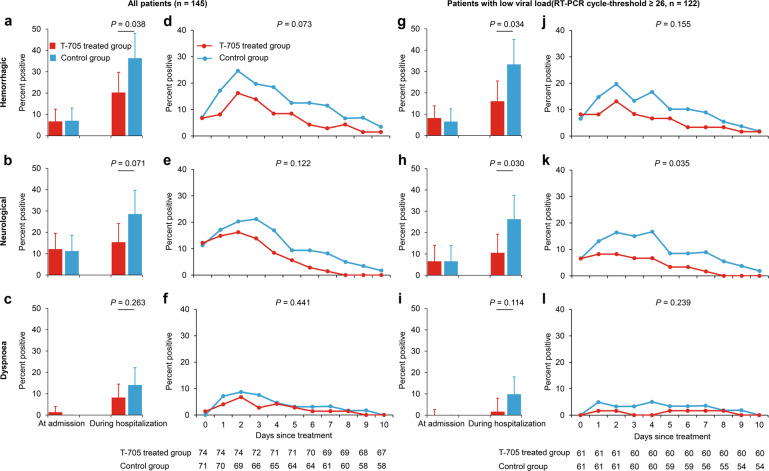
The percentage of patients presenting severe complications. **a**–**c**, **g**–**i** The incidences of severe complications at admission and during hospitalization. The percentages of patients presenting hemorrhagic signs (**a**, **g**), neurological symptoms (**b**, **h**), and dyspnea (**c, i**) are shown at each time point by treatment arm for all patients (*n* = 145) and for patients with low-baseline viral loads (RT-PCR cycle-threshold value ≥26, *n* = 122). The bars indicate the 95% confidence intervals (only the upper half is shown). **d**–**f**, **j**–**l** The daily percentage of severe complications in the patients. The mean percentages of neurological symptoms, hemorrhagic signs, and dyspnea are shown over time for all the patients (**d**–**f**) and the patients (**j**–**l**) with low-baseline viral loads (RT-PCR cycle-threshold value ≥26). The numbers of patients who contributed to the at-risk population at each time point are shown under the *x* axis. The difference of these severe complications was analyzed over time (the curves) by using the generalized estimating equation model

**Fig. 7:**
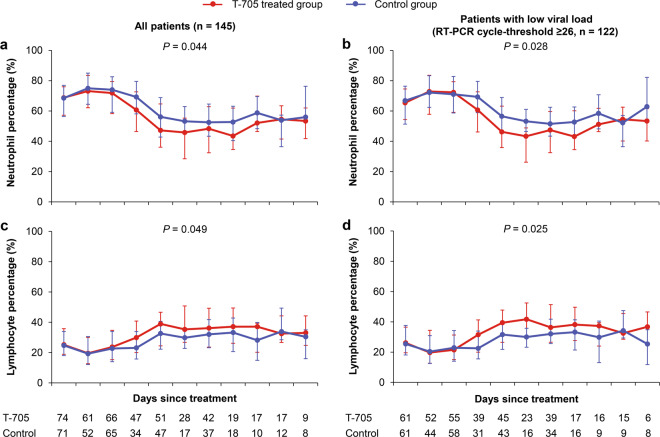
The kinetics of key laboratory parameters in the patients. The median (quartile range) values of neutrophil percentage and lymphocyte percentage are shown over time for all the patients (*n* = 145, **a** and **c**), and the patients (*n* = 122, **b** and **d**) with low-baseline viral loads (RT-PCR cycle-threshold value ≥26). The numbers of patients who contributed to the at-risk population at each time point are shown under the *x* axis. The difference of these severe complications was analyzed over time (the curves) by using the generalized estimating equation model

The development of severe complications (hemorrhagic signs, neurological symptoms, and dyspnea) and the daily measurements of laboratory parameters (aspartate aminotransferase (AST), lactate dehydrogenase (LDH), creatine kinase (CK), platelet (PLT) count, neutrophil percentage, and lymphocyte percentage) that are known to predict fatal outcome^4^ were also analyzed as other secondary outcomes. T-705 treatment reduced the occurrence of hemorrhagic signs in all patients as well as in the subgroup of low-baseline viral loads (*P* = 0.038 and *P* = 0.034, respectively; Fig. 6a, g). The occurrence of neurological symptoms was reduced by the T-705 treatment only among patients with low-baseline viral loads (*P* = 0.030; Fig. 6h). The incidence of dyspnea was not affected by T-705 treatment regardless of the initial viral load (both *P* > 0.05; Fig. 6c, i). Based on the GEE models for daily presence/absence of clinical manifestations, all three severe complications resolved more rapidly in T-705-treated patients (Fig. 6d–f, j–l); however, only showing a significant difference for neurological symptoms among the patients with low-baseline viral loads (*P* = 0.035; Fig. 6k). T-705 treatment also significantly expedited the recovery of neutrophils and lymphocytes to normal in all patients (*P* = 0.044 and *P* = 0.049, respectively; Fig. 7a, c), as well as in the subgroup of patients with low-baseline viral loads (*P* = 0.028 and *P* = 0.025, respectively; Fig. 7b, d), while did not show the effect on the recovery of PLT count, AST, LDH, and CK (All *P* > 0.05; Supplementary Fig. S11).

### Safety evaluation of T-705 treatment in SFTS patients

The dosage of T-705 in this clinical trial was lower than that used in treating Ebola virus disease (EVD), which showed well-tolerance in human.^35^ Generally, we did not observe severe adverse events during hospitalization for both treatment arms, except for skin allergy that was seen in one patient after receiving T-705 treatment. Although from day 2 to day 9 after treatment initiation, higher uric acid levels were indeed observed in the T-705-treated group than in the control group, the mean level did not exceed the upper limit in males or females (Supplementary Fig. S12). All survivors were asked to return for clinical assessment when 1 month after discharge from the hospital to evaluate the long-lasting effect. From either group of participants, except for one T-705-treated patient showing a slightly increased serum level of LDH, no laboratory abnormality was observed, neither was SFTSV RNA detected. Together, these results suggested that T-705 showed treatment benefit for SFTS patients, especially for those with low-baseline viral loads, and did not pose severe adverse effects.

### Antiviral mechanism of T-705 on SFTSV replication in patients

**Fig. 8:**
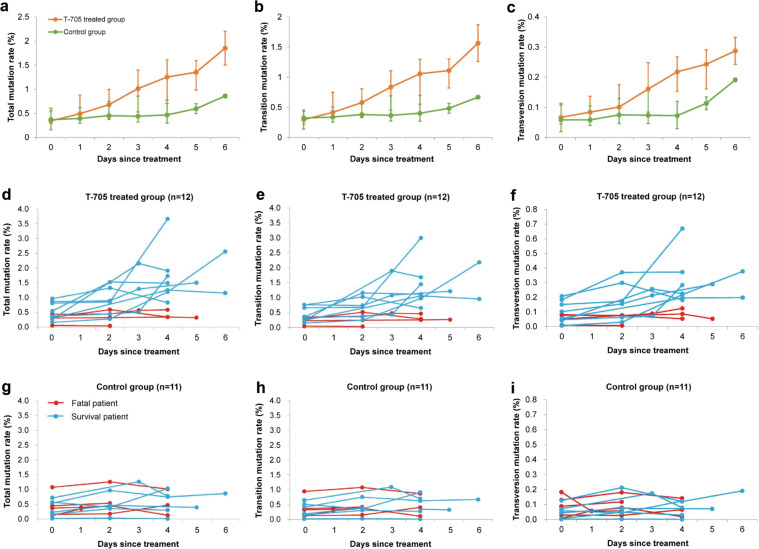
The kinetics of the viral mutation in SFTS patients. A total of 40 samples were obtained from 12 patients receiving T-705 treatment (4 fatal and 8 survival) and 34 samples were obtained from 11 controls (5 fatal and 6 survival). The mean total mutation rates, transition mutation rates, and transversion rates, and ratio of transitions/transversions are shown over time for the two groups (**a**, **b**, **c**). The daily value of total mutation rate, transition mutation rate, and transversion rate was shown over time for each patient in the T-705 treated group (**d**, **e**, **f**) and the control group (**g**, **h**, **i**)

As to human patients, the mutagenesis pattern of T-705 treatment has only been reported in a man with norovirus infection.^36^ To determine the potential mutagenesis mechanism of action of T-705 in SFTSV infected patients, we performed NGS and mutation analyses on 74 serum samples longitudinally collected from 12 T-705-treated patients and 11 controlled patients (Fig. 8), with a mean (±SD) sequencing depth of 3090 (±840) fold genome coverage obtained. Before treatment, the rates of total, transition, and transversion mutations were comparable between the two groups (All *P* > 0.05; Supplementary Table S3). During the first week of treatment, all the mutation rates were significantly increased in the T-705 treated group in comparison with the control group, with *P* values of 0.035, 0.041, and 0.019 for total, transition, and transversion mutations respectively (Fig. 8a–c), while the ratio of transitions/transversions did not show a significant difference between the two groups (*P* > 0.05, GEE model; Supplementary Fig. S13). The most pronounced difference (defined as a change of mutation rate from baseline) was observed at day 4 (Table 2). Increased virus mutations were obviously seen in the survival patients but not seen in the fatal patients after the treatment with T-705 (Fig. 8e–g), while no increase of virus mutations was observed in survival or fatal patients without receiving T-705 treatment (Fig. 8i–k). The dynamic trend of accumulating mutations displayed a consistent pattern with that of the CT values of SFTSV in the T-705 treated group (Supplementary Fig. S14), suggesting that T-705 reduces the viral loads likely through inducing both transition and transversion mutations that are detrimental to viral proliferation. As seen in the mouse model, no predominant pattern of transition mutations or transversion mutations was observed in serum samples collected from SFTS patients (Supplementary Fig. S15).

**Table 2:** Comparison of the changes of mutation rates from baseline between the T-705-treated group and the control group at days 2 and 4 since treatment

	Days since treatment	Changes from baseline		Difference between two groups	*P* value
		Control group	T-705-treated group		
Total mutation rate, mean (CI 95%), %	2	0.14 (0.02 to 0.26)	0.19 (−0.03 to 0.41)	0.05 (−0.18 to 0.28)	0.663
	4	0.14 (−0.01 to 0.29)	0.89 (0.22 to 1.55)	0.75 (0.08 to 1.42)	0.033
Transition mutation rate, mean (CI 95%), %	2	0.11 (0.01 to 0.21)	0.15 (-0.03 to 0.33)	0.04 (−0.15 to 0.23)	0.675
	4	0.12 (−0.01 to 0.26)	0.74 (0.18 to 1.29)	0.61 (0.05 to 1.17)	0.035
Transversion mutation rate, mean (CI 95%), %	2	0.02 (−0.03 to 0.06)	0.04 (-0.01 to 0.08)	0.02 (-0.03 to 0.08)	0.375
	4	0.02 (−0.01 to 0.04)	0.15 (0.04 to 0.27)	0.13 (0.02 to 0.25)	0.029

## Discussion

T-705 has been shown to be converted to T-705-4-ribofuranosyl-5-triphosphate (T-705RTP) by intracellular enzymes.^37^ T-705RTP is recognized as a purine nucleotide by viral RNA-dependent RNA polymerase (RdRp), which inhibited the incorporation of CTP and UTP and disrupted the fidelity of the RdRp, leading to base mismatches and further loss of fitness without generation of truncated genomes.^38^ During RNA virus replication, error rates of RNA genome are finely balanced to generate ample diversity while maintaining sufficient accuracy in the transmission of genetic information, and slight increases in virus mutation frequencies might result in extinction.^34^ Recent studies indicated that T-705 treatment induces an increase in mutagenesis in various RNA viruses, including EBOV,^24,25^ influenza virus,^28,38^ norovirus,^34^ resulting in a significant reduction in the viral-specific infectivity and hence in extinction. The current study, based on results from in vitro and in animal experiments, as well as clinical samples, demonstrated that T-705 treatment led to increases in mutation rates of the SFTSV genome that was related to the reduced viral loads, suggesting that the anti-SFTSV activity of T-705 was mediated by inducing mutagenesis. Unlike in vitro experiments, our findings from the animal experiments and clinical samples revealed that T-705 treatment could induce both transition and transversion mutations. Transversion substitutions that are more likely to induce nonsynonymous have been thought to be more detrimental to RNA viruses including influenza virus and HIV,^39^ therefore, the increased transversion mutations may be associated with the suppression of SFTSV. Moreover, we also found that T-705 had a more significant mutagenic effect on L and M segments. Considering the important role of RdRp (encoded by L) and G proteins (encoded by M) in SFTSV replication, the mutagenesis in L and M segments may be more likely to result in the extinction of the virus.

In this study, we reported the first randomized controlled trial evaluating the potential therapeutic effect of T-705 against viral infection, SFTSV infection in this case. The CFR of SFTS patients was reduced among those assigned to T-705 plus the standard supportive care than those assigned to the standard supportive care (9.5% vs. 18.3%), which corresponded to a 46.6% lower relative risk of fatal with T-705 treatment, although showing no statistical significance. However, after adjusting the variables that might affect the disease fatal outcome by performing multivariate analysis, we revealed a significant reduction in CFR attained by T-705 therapy. The patients receiving T-705 treatment needed a shorter time to clear SFTSV, and presented less hemorrhagic signs and more rapid recovery of neutrophils and lymphocytes. In addition, we did not observe evidently severe adverse events during T-705 treatment. Higher uric acid levels were observed in the T-705-treated group than the control group, but the mean level did not exceed the upper limit in males or females. Since leukocytopenia, thrombocytopenia, and elevated liver enzyme levels are important clinical features of SFTS, we cannot infer their causal relationship to T-705 drug use. Nonetheless, there was no significant difference in these parameters between the two arms, when were evaluated as secondary outcomes. Still, it is logical to conclude that no such side effects with T-705 were observed in this study.

Of importance, a remarkable reduction in CFR with T-705 (1.6% vs. 11.5%) was shown in the subgroup with low-baseline viral load (RT-PCR CT ≥ 26). Likewise, for this subgroup, a distinct effect of T-705 on reducing the occurrence of severe complications, accelerating SFTSV clearance, and restoring laboratory abnormalities was observed. This subgroup accounted for over 80% of all SFTS patients, suggesting a promising potential of T-705 in treating SFTS, especially when compared with ribavirin that was effective in about only 20% of all patients.^4^ Our in vivo analysis on the mouse model here also found T-705 increased the survival rate of SFSTV-infected mice significantly while ribavirin did not have such an effect. These clinical findings corroborate a recent report that ribavirin was less effective for treating SFTSV infection than T-705 both in vitro and in animal model.^31^ Nonetheless, it needs to be mentioned that the statistical power of the subgroup analysis was 62% based on the current sample size (61 in each group) at a significance level of 0.05. If the T-705 treatment effect sustained and the sample size reached 100 per group, the statistical power would be 83%.

For the patients with high-baseline viral loads, the CFR was moderately reduced in the T-705-treated group (46.2% vs. 60.0%). The lack of statistical significance could be due to both the small sample size and the relatively low drug doses. Indeed, our in vivo study showed that T-705 treatment efficacy was affected not only by the concentration and action time of the drug but also by the viral loads. Previous pharmacokinetics analysis for the JIKI trial indicated that concentrations of T-705 in patients infected with Ebola virus were lower than targeted, which might have led to the underestimation of the antiviral efficacy of T-705.^40,41^ Following a previous study,^42^ the dose here was set as 1800 mg twice on the first day and 1000 mg twice during the following days, which was estimated to attain a median *C*_ave_ not lower than 50 μg/ml, the effective dose in an anti-influenza randomized clinical trial.^19^ This dose, however, is slightly lower than that used in the trial of treating the Ebola virus disease.^41^ Further pharmacokinetics analysis is needed to evaluate the actual anti-SFTSV effect under various T-705 concentrations.

Our study proved in an explicit manner, the fatal outcome was seen in patients who presented less mutagenesis, despite receiving T-705 treatment. It is reasonable to hypothesize the mutagenesis evaluation of SFTSV might be more direct and effective in predicting the disease outcome. There was an obvious difference between fatal and survived patients for their sensitivity to T-705, suggesting T-705 induced mutagenesis was highly dependent on the initial viral loads. As had previously shown for the foot-and-mouth disease, successful viral clearance was often associated with lower initial viral loads.^43^ It needs to be disclosed whether SFTS patients with high viral load could get more treatment benefit if receiving the higher dose of T-705.

Besides, death occurred on T-705-treated patients, an unexpected outcome that might result from excessive inflammation response. As part of the immunological measurements in the current research, IL-10, IL-6, IFNA-γ, TNF-α, and GM-CSF were all remarkably elevated in fatal patients than survivors (Supplementary Fig. S16), playing a crucial role in determining the final disease outcome, irrespective of the viral loads. As such, the therapeutic regimens for SFTSV infection should also regulate the host inflammatory response, which may hold promise in preventing the death outcome.

There is an utmost need for potent antivirals to combat the infections of emerging and high pathogenic viruses. The clinical efficacy of T-705 has been reported in treating Ebola virus and Lassa virus infections.^24,44,45^ As to SFTS that has been prevalent in East Asia for almost 10 years, until very recently, there is only one case report describing the successful treatment of two SFTS patients with complete remission by using T-705.^46^ Taken this together, the current study represented the first randomized clinical trial showing T-705 is both safe and effective in treating SFTS, holding the promise of curing the patients, especially for those with a baseline CT value ≥26. It is well documented that T-705 has broad antiviral activity against RNA viruses in vitro or in animal models. Therefore, the use of T-705 would allow considering a large number of these RNA viruses as potential therapeutic targets. Beyond all the clinical significance, the elucidation of the antiviral mechanism of T-705 paved the way for future clinical trials evaluating T-705 on other RNA viruses, for example, the ongoing epidemic of heartland virus that inflict more and more cases in the USA. The current research also provided a dose regimen choice of T-705 in treating patients with other emerging infectious diseases, e.g., the still-expanding SARS-CoV-2 around the world in a context where trials are urgently needed.

In conclusion, our study showed that administration of T-705 can reduce case fatality in SFTS patients, possibly via inducing lethal mutagenesis, and such mutagenesis may be mediated by drug dose and viral load. Although designed as a randomized clinical trial, the single-blind design was applied due to the high mortality of the infection. This weakness might potentially elicit observational bias at the bedside, as opposed to a double-blind design. Still, our findings warrant verification by further clinical trials of larger scales. A T-705-ribavirin combine treatment, which has reached a higher efficacy through a potentially synergistic antiviral effect in treating Lassa fever and Crimean-Congo hemorrhagic fever,^45,47^ should also be advocated in future clinical trials.

## Materials and methods

### In vitro experiments

Vero cells pre-seeded in 24-wells plate were inoculated with 200 μl of SFTSV strain HNXY2017-66 at the multiplicity of infection (MOI) indicated and incubated for 1 h at 37 °C. Supernatants were removed, and cells were washed with PBS to remove the unattached virus, and fresh media containing T-705 or DMSO (vehicle) were added into each well. At 24 h post infection, both cell and supernatant were collected. For experiments involving the serial passage of virus in the presence of T-705, passage 1 (P1) cells were infected with SFTSV and treated with T-705. In subsequent passages, 200 μl supernatant of the previous passage (1/5 of the total virus) was added to a new monolayer of Vero cells. A total of four passages (P1–P4) were performed, and both cells and supernatant were collected (Supplementary Fig. S1). Another in vitro experiment involving different MOIs of SFTSV was performed as described in Supplementary Fig. S3. Viral titers in supernatants were determined by immunological focus assay on Vero cells, as previously described.^48^ Total RNAs were extracted from both cell and supernatant and subjected to quantitative RT-PCR and NGS analysis. For quantitative RT-PCR, RNAs were subjected to reverse transcription using PrimeScript RT reagent Kit with gDNA Eraser (TaKaRa). Quantitative real-time PCR was performed with SYBR Premix Ex Taq (Applied Biosystems) on an Applied Biosystems 7500 real-time PCR system. For NGS analysis, samples were prepared using NEBNext Ultrat Ultra prepared uem.CR sysIllumina (NEB). Refer to the Supplementary Materials and Methods for a further description of the in vitro experimental procedures.

### Animal studies

Six to nine-week-old IFNAR^−/−^ C57BL/6 mice were kept in an environmentally controlled specific-pathogen-free (SPF) animal facility in the Laboratory Animal Center of Academy of Military Medical Sciences (Beijing, China). Forty-six IFNAR^−/−^ C57BL/6 mice were used to examine the treatment effect of T-705 and ribavirin on the probability of survival, with detailed descriptions shown in Supplementary Materials and Methods.

For mutation analysis of T-705 treatment in vivo, 27 6–9-week-old female IFNAR^−/−^ C57BL/6 mice were divided into three groups (nine mice per group): SFTSV + vehicle group, SFTSV + T-705 group I (300 mg/kg/d), and SFTSV + T-705 group II (150 mg/kg/d) (Supplementary Fig. S16). In infection experiments, mice were intraperitoneally inoculated with 10^3^ FFU of SFTSV strain HNXY2017-66 in 100 μl DMEM, or the same volume of DMEM. T-705 was dissolved in PBS and given by using a stomach probe. Treatments were commenced 1 h post infection and continued for 4 days. Three IFNAR^−/−^ C57BL/6 mice from each group were sacrificed on days 2, 3, and 4 p.i., respectively. Prior to sacrifice, blood samples were collected via cardiac puncture from anesthetized mice and used for serum isolation. Spleen samples were subsequently collected. RNA was extracted from serum and spleen samples with the use of Purelink Viral RNA/DNA Kit (Invitrogen) and RNeasy Mini Kit (QIAGEN). A portion of the RNA was subjected to quantitation of SFTSV RNA by qRT-PCR as above described. The remaining RNA was subjected to one-step reverse transcription PCR with PrimeScript^TM^ One-Step RT-PCR Kit (TaKaRa) using primers spanning the whole viral genome, and the resultant PCR products were purified and prepared for NGS using TruePrepTM DNA Library Prep Kit V2 (Vazyme).

### Clinical study design and participants

This investigator-initiated, prospective, single-blinded, and randomized controlled trial was conducted at the People’s Liberation Army (PLA) 154 hospital, Xinyang, Henan province, China. Xinyang city located at the center of the Dabie mountains which is the area of greatest SFTS endemicity and the PLA 154 hospital received the majority of SFTS patients in this area.^18^ The study protocol was approved by the hospital’s ethics committee before the start of the study (number 154YY-LL-2018-02). All procedures were in full compliance with the Declaration of Helsinki and the principles of Good Clinical Practice and the protocol was registered on the Chinese Clinical Trial Registry website (number ChiCTR1900023350).

Subjects first underwent a physical examination and routine laboratory tests (hemogram and biochemical blood analysis) when being admitted to the hospital. Those who met the case definition for clinically diagnosed SFTS patients according to the criteria released by the Chinese Ministry of Health,^49^ had their serum samples collected at admission and subjected to detection of SFTSV RNA by reverse transcriptase-polymerase chain reaction (RT-PCR), with detailed description seen Supplementary Materials and Methods. Patients with positive SFTSV RNA detection were defined as laboratory-confirmed SFTS, and further interviewed by healthcare clinicians using a standardized questionnaire to assess their eligibility into the study (Supplementary Table S4). Written informed consent was signed before the questionnaire. Exclusion criteria included patients aged <18 years, pregnant or lactational women, patients with chronic diseases (i.e., cancer, acquired immunodeficiency syndrome, diabetes, hepatitis, and pulmonary tuberculosis), patients infected with other vector-borne pathogens (e.g., *Rickettsia* sp., *Borrelia* sp., and *Babesia* sp.), patients complicated with underlying diseases (i.e., hematologic, renal, hepatic, or autoimmune dysfunction), patients having a history of hypersensitivity to an antiviral nucleoside-analog drug targeting a viral RNA polymerase, patients currently using adrenocorticosteroids (except topical preparation) or immunosuppressive drugs, and patients with contraindication for the use of T-705 (a history of gout and hyperuricemia).

### Randomization, masking, and procedures

The eligible patients who agreed to have their samples and information collected were enrolled in the study. Patients were immediately randomly assigned by using a random number list generated by Microsoft Excel program (version 2013) in a 1:1 ratio, to receive the standard supportive care (the control group) or the standard supportive care combined with T-705 (the T-705-treated group). The researcher who allocated patients did not take charge of patients’ enrollment. Only patients were masked to the assigned drugs.

Treatment was generally initiated within 24 h after randomization. The administered T-705 was produced by the Beijing Institute of Microbiology and Epidemiology and approved by the Ministry of Health of the PLA (approved number FPT0501). Patients allocated to the T-705-treated group received tablets of 1800 mg T-705 orally twice in the first day (3600 mg total), and tablets of 1000 mg twice on day 2 lasting at least 5 days or until to serum SFTSV RNA concentration was reduced below the low limit of quantification or until to the patients were discharged from the hospital. All other therapeutic decisions were at the discretion of the primary physician but were constrained to standard supportive care.

Blood samples were collected for the assessment of serum SFTSV RNA concentrations with the use of RT-PCR during the patients’ hospitalization. Routine laboratory tests, including hemogram and biochemical blood analysis, were prescribed at least every other day during hospitalization, and the results were reported by the medical clinical laboratory of the PLA 154 hospital (Supplementary Table S5). Clinical manifestations, including signs/symptoms related to SFTSV infection and adverse effects related to the administration of T-705, were recorded daily on a structured case report form by doctors and nurses on duty until their signs/symptoms resolved or their viremia turned negative or they were discharged from the hospital (Supplementary Tables S6 and S7).

### Outcomes

The primary outcome was case fatality. The fatal outcome was firstly retrieved from medical records and further verified by performing a follow-up visit. The follow-up was required for all patients who discontinued therapy or had been discharged from hospital because of adverse clinical progression or for economic reasons, which was completed within 1 week of discharge by a phone call or home visit to determine their final outcome (death or survival). The secondary outcomes included the time needed for viral clearance (defined as the time to a first negative RT-PCR assay) and dynamic changes of viral load during hospitalization, the development of severe complications (hemorrhagic signs, neurological symptoms, and dyspnea), and the daily measurements of laboratory parameters (aspartate aminotransferase (AST), lactate dehydrogenase (LDH), creatine kinase (CK), platelet (PLT) count, neutrophil percentage, and lymphocyte percentage) that are known to predict fatal outcome.^4^

Due to most of the adverse effects related to the administration of T-705, including gastrointestinal symptoms (i.e., nausea, vomiting, abdominal pain, and diarrhea), leukocytopenia, thrombocytopenia, and elevated liver enzyme levels, resembled the clinical features of SFTSV infection and were generally mild, we only compared severe manifestations between the two groups for safety assessments, which comprised allergy, jaundice, toxic epidermal necrolysis, mucocutaneocular syndrome, and hemorrhagic colitis. Besides, serum uric acid levels related to the administration of T-705 were also monitored and compared between the two groups.

### Statistics

According to our previous observational clinical study, the average case fatality rate of laboratory-confirmed SFTS patients from May to August was about 17%.^4^ With the aim of testing a relative difference of 85% in case fatality rate between the two groups at a significance level of 0.05 with a statistical power of 85%, we determined the minimal sample size to be 70 for each group.

All patients who had primary outcome recorded were included in the primary efficacy analysis. The differences of baseline data between two groups were assessed by the two-sample *t* test or the nonparametric Mann–Whitney test for continuous variables, and by the chi-square test or the Fisher’s exact test for categorical variables. The choice of the test depended on the distributional characteristics of the data. For example, the Mann–Whitney test was used for highly skewed variables.

The Kaplan–Meier survival curves were plotted for time-to-event data and the log-rank test was used to compare treatment arms. Given the previous findings that age and delay from symptom onset to hospital admission were associated with fatal outcome of SFTS,^4,16^ these two factors, together with gender, were therefore adjusted by performing Cox proportional hazards model to fit time-to-event data and estimate hazard ratios (HRs) and 95% confidence intervals (CIs) for treatment. The two-sample *t* test was applied to compare the time needed for viral clearance between the two groups. The chi-square test or the Fisher’s exact test was applied to compare the frequency of severe complications that were developed during treatment between the two groups. A generalized estimating equation (GEE) model was used to analyze the data on daily measurements of viral loads and laboratory parameters.

Post hoc subgroup analyses were performed for the primary outcome with regard to baseline viral load, the delay from symptom onset to hospital admission, age, and gender. Considering that the baseline viral load affects the treatment benefit, post hoc subgroup analyses were also performed for the secondary outcomes. A two-sided *P* value <0.05 was considered statistically significant. All analyses were performed using the SPSS software (version 19.0).

### Sample collection of SFTS patients for mutation analysis

Serial serum samples were collected from the SFTS patients who were included in the clinical trial and subjected to RNA extraction. Total RNAs were used for viral genome amplification and then prepared for NGS using TruePrepTM DNA Library Prep Kit V2 (Vazyme).

### Next-genome sequencing and mutation analysis

Next-genome sequencing was completed on an Illumina MiSeq generating a 150-bp paired-end read. Reads were mapped with BWA v0.7.5 and converted to BAM files using SAMTools (1.1.2).^50^ A de novo contig was produced to obtain a consensus sequence using Galaxy (https://mississippi.snv.jussieu.fr/), and a consensus sequence was manually checked. Samples were compared with reference sequence or consensus sequence using bwa mem, and mutation analysis was performed with samtools mpileup. The volume of variant nucleotides per genome took account of the frequency of mutations at each variant site. Quantification of mutants was reported as the relative percentage of distinct genomic variant sites in a given biological sample. Analyses included the characterization of the variants (transitions/transversions) and the changes of the percentage of mutations over time. Transition indicates purine to purine mutation (G,A → A,G) or pyrimidine to pyrimidine (T,C → C,T) mutations, while transversion indicates purine to pyrimidine mutation (G,A → C,T) or pyrimidine to purine (T,C → G,A) mutations.

## Supplementary information

Supplementary_Materials

## Data Availability

The datasets in this study are available from the corresponding author upon reasonable request.
