# Supplementary material for: Clinical effect and antiviral mechanism of T-705 in treating severe fever with thrombocytopenia syndrome
Source: Signal Transduct Target Ther. 2021 Apr 16;6:145. doi: 10.1038/s41392-021-00541-3 (PMC8050330; doi:10.1038/s41392-021-00541-3)
Supplement: Supplementary file 1 — Supplementary_Materials [file 41392_2021_541_MOESM1_ESM.docx]

Supplementary Materials for

**Clinical effect and antiviral mechanism of T-705 in treating severe fever with thrombocytopenia syndrome**

Hao Li,^1†*^ Xia-Ming Jiang,^2*^ Ning Cui,^3^ Chun Yuan,^3^ Shao-Fei Zhang,^1^ Qing-Bin Lu,^4^ Zhen-Dong Yang,^3^ Qin-Lin Xin,^2^ Ya-Bin Song,^1^ Xiao-Ai Zhang,^1^ Hai-Zhou Liu,^2^ Juan Du,^4^ Xue-Juan Fan,^3^ Lan Yuan,^3^ Yi-Mei Yuan,^3^ Zhen Wang,^3^ Juan Wang,^3^ Lan Zhang,^3^ Dong-Na Zhang,^1^ Zhi-Bo Wang,^1^ Ke Dai,^1^ Jie-Ying Bai,^5^ Zhao-Nian Hao,^6^ Hang Fan,^1^ Li-Qun Fang,^1^ Gengfu Xiao,^2^ Yang Yang,^7^ Ke Peng,^2^ Hong-Quan Wang,^1†^ Jian-Xiong Li,^9†^ Lei-Ke Zhang,^2†^ Wei Liu^1,8†^

Correspondence to: liuwei@bmi.ac.cn, lwbime@163.com; zhangleike@wh.iov.cn; 301301ljx@sina.com; wanghq@bmi.ac.cn; lihao_1986@126.com

**This file includes:**

Materials and Methods

Supplementary Text

Figures. S1 to S16

Tables. S1 to S57

References

**Materials and Methods**

***In vitro* experiments**

***Cells and viruses***

Vero cells were obtained from the American Type Culture Collection (ATCC), and grown in Dulbecco’s modified Eagle’s medium (DMEM; Gibco) supplemented with 10% fetal bovine serum (FBS; Gibco), 100 U/ml of penicillin, and 100 μg/ml of streptomycin (Gibco) at 37°C with 5% CO_2_.

SFTSV strain HNXY2017-66 was isolated from the serum from patients in Chinese People’s Liberation Army 154 hospital in Xinyang city of Henan Province, and propagated in Vero cells. Viral titer was determined by immunological focus assay on Vero cells. Briefly, confluent monolayers were incubated with 10-fold dilutions of virus for 1 hour, and then culture medium was replaced by DMEM containing 2% serum and supplemented with 1.1% carboxymethyl-cellulose. Foci were visualized by two-step immunostaining with an antibody against viral protein NP and an anti-rabbit horseradish peroxidase-conjugated secondary antibody (Proteintech).

***Quantitative real-time PCR analysis of intracellular SFTSV RNA***

Cells were harvested, and total cellular RNA was extracted using TRIzol reagent (Promega). Then RNA was subjected to reverse transcription using a Moloney murine leukemia virus (MMLV) reverse transcriptase (Promega). Quantitative RT-PCR was performed using specific primers for SFTSV and GAPDH with SYBR Premix Ex Taq (Applied Biosystems) on an Applied Biosystems 7500 real-time PCR system.

***Quantitative real-time PCR analysis of supernatant SFTSV RNA***

Supernatants were harvested and the total RNA was extracted by using the QIAamp Viral RNA Mini Kit (Qiagen). For quantitation of vRNA, a standard curve was constructed with a plasmid containing full-length nucleotides of the S gene of the respective virus, and qRT-PCR was performed with the One-step Primer Script RT-PCR Kit (Takara) in a LightCycler 480 (Roche).

***MTT assay***

Cells pre-seeded in 96-well plates were treated with the desired concentrations of compound for 24 h, and 3-(4,5-dimethylthiazol-2- thiazolyl)-2,5-diphenyl-2H-tetrazolium bromide (MTT; Sigma-Aldrich) was added at a final concentration of 5 mg/ml. The cells were incubated at 37°C for 4 h, and the supernatant was removed. Then 50 μl of DMSO was added to each well, and the emitted light at 492 nm was measured using a Thermo Multiskan enzyme-linked immunosorbent assay (ELISA) reader (Thermo).

**Animal studies**

***T-705 treatment effect on probability of survival in IFNAR^-/-^ C57BL/6 mouse***

Six to nine-week-old IFNAR^-/-^ C57BL/6 mice were kept in an environmentally controlled specific-pathogen-free (SPF) animal facility in the Laboratory Animal Center of Academy of Military Medical Sciences (Beijing, China). According to the sex and weight before the inoculation of virus, the IFNAR^-/-^ C57BL/6 mice were divided into five groups: SFTSV + vehicle group (5 female and 5 male mice), SFTSV + T-705 group I (300 mg/kg/d, 5 female and 5 male mice), SFTSV + T-705 group II (150 mg/kg/d, 5 female and 5 male mice), SFTSV + ribavirin (100 mg/kg/d, 5 female and 5 male mice), and DMEM + T-705 group (300 mg/kg/d, 3 female and 3 male mice). In infection experiments, mice were intraperitoneally inoculated with 10^3^ FFU of SFTSV strain HNXY2017-66 in 100 μl DMEM, or the same volume of DMEM. SFTSV strain HNXY2017-66 was isolated from the serum of a SFTS patient in Chinese People’s Liberation Army 154 hospital in Xinyang city of Henan Province, and propagated in Vero cells. T-705 was dissolved in PBS and given by using a stomach probe. Treatments were commenced 1hour post infection and continued for 5 days. The mice were monitored daily for signs of disease (hunched posture, ruffled fur, decreased activity, and response to stimuli) and body weight after infection, and when criteria for euthanasia were fulfilled, the animals were euthanized with an isoflurane overdose followed by cervical dislocation. Serum and spleen were then collected. Total RNA was extracted by using the QIAamp Viral RNA Mini Kit (Qiagen) and subjected to qRT-PCR by using the One-step Primer Script RT-PCR Kit (Takara) in a LightCycler 480 (Roche). Animal experiment was performed in accordance with the National Institutes of Health guidelines under protocols approved by the institute’s Animal Care and Use Committee.

**Clinical study**

***Diagnosis criteria of SFTS***

According to the case definition released by the Chinese Ministry of Health[^1^](#_ENREF_1), suspected SFTS was defined as meeting the following criteria: (1) exposure history (field activities in SFTS-endemic areas or tick bites within 2 weeks); (2) presentation of acute fever (temperature, of ≥37.5°C); (3) laboratory abnormalities including thrombocytopenia (platelet count, <100 × 10^9^ platelets/L) and/or leukopenia (leukocyte count, <4.0 × 10^9^ leukocytes/L). Laboratory-confirmed SFTS was defined as meeting 1 or more of the following criteria: (1) detection of SFTSV RNA by a molecular method; (2) isolation of SFTSV in cell culture; (3) seroconversion or ≥4-fold increase of antibody titers between 2 serum samples collected at least 2 weeks apart.

***Detection of SFTSV RNA in serum samples of SFTS patients by reverse transcriptase polymerase chain reaction***

Serum samples were collected from clinically diagnosed patients at admission and during hospitalization. Viral RNA was isolated from serum samples using QIAamp Viral RNA Mini Kit (Qiagen), according to the manufacturer’s instructions. One step Primer Script RT-PCR Kit (TaKaRa) was used according to the manufacturer’s instructions for SFTSV detection in a volume of 20 µl containing 10 µl of One Step RT-PCR Buffer (2×), 0.4 µl of TaKaRa Ex Taq HS (5 U/µl) and 0.4 µl of PrimeScriptTM RT Enzyme Mix II, 1 µl of PCR primer mix (20 µM of sense and antisense each) and 0.5µl of Probe (10 µM ), total RNA 2 µl and RNase free dH2O (5.7 µl). PCR was carried with one cycle of 42 ºC for 5 min and 95ºC for 10 sec, followed by 40 cycles of 95ºC for 5 sec and 55ºC for 20 sec in a Mini-8 Real-Time PCR System (Coyote Bioscience, Beijing, China). The real-time PCR primers and probe targeting at the S-segment of the SFTSV were described as previously[^2^](#_ENREF_2).

**Supplementary Text**

**Protocol for the single-blind, randomized controlled trial to assess the efficacy and safety of T-705 in treating SFTS (Chinese Clinical Trial Registry website, number ChiCTR1900023350).**

**1. Introduction**

Severe fever with thrombocytopenia syndrome (SFTS), an emerging infectious disease caused by a bunyavirus (SFTS virus, SFTSV), was first discovered in China in 2009[^3^](#_ENREF_3). Common clinical features include acute fever, leucocytopenia, thrombocytopenia, feeble, myalgias, and gastrointestinal symptoms. Patients with severe illness usually present hemorrhagic signs, neurologic symptoms, and multiple organ failure[^4^](#_ENREF_4). Until to Dec 2014, a total of 2000 SFTS cases have been reported in 19 provinces in China, with a high case fatality rate ranging from 7% to 30%[^5^](#_ENREF_5). Among the 19 provinces, Henan province reported the greatest number of SFTS cases, with a proportion of 48%. SFTS was subsequently reported in South Korea and Japan in 2012 [^6^](#_ENREF_6)^,^[^7^](#_ENREF_7). Tick-to-human transmission is the primary infection route, with Haemaphysalis longicornis tick as the predominant vector of SFTSV ^[8](#_ENREF_8)^. Human-to-human transmission has also been reported, and the most common risk of the transmission is direct blood exposure [^9^](#_ENREF_9)^,^[^10^](#_ENREF_10). All these findings raised the possibility of pandemic transmission of SFTSV outside of Asia, and the high risk of human-to-human transmission other than through blood.

The reported treatment options for SFTS included ribavirin, corticosteroid therapy, and plasma exchange, but there is no approved specific therapy [^2^](#_ENREF_2)^,^[^11^](#_ENREF_11). The neutralizing antiserum collected from the recovery period of SFTS patients showed some therapeutic effects in mouse model, but the source of antiserum is limited. Ribavirin also showed limited therapeutic effects in mouse model. A retrospective study reported that ribavirin was ineffective for improving the disease outcome [^2^](#_ENREF_2), and an observational study revealed that ribavirin showed limited efficacy when used early among patients with very low viremia [^12^](#_ENREF_12). Investigation on more effective antiviral therapies became urgent as the epidemics of SFTS continue.

Favipiravir (T-705) is a novel viral RNA polymerase inhibitor and is phosphoribosylated by cellular enzymes to its active form, favipiravir-ribofuranosyl-5′-triphosphate. Its antiviral effect is attenuated by the addition of purine nucleic acids, indicating the viral RNA polymerase mistakenly recognizes favipiravir-RTP as a purine nucleotide. Differing from ribavirin that acts by GTP depletion via inhibition of IMP dehydrogenase, T-705 functions as a GTP-competitive inhibitor of the viral polymerase [^13^](#_ENREF_13)^,^[^14^](#_ENREF_14). The compound shows strong inhibition activity against a broad spectrum of RNA viruses in vitro or in animal models, including flaviviruses (yellow fever virus and West Nile virus), arenaviruses (Junin virus and Machupo virus), filoviruses (Ebola virus), and some members in the family of bunyaviridae (i.e. Rift Valley Fever virus, Sandfly fever virus, and Punta Toro virus) [^15-18^](#_ENREF_15). A report showed that T-705 inhibited SFTSV replication both in vitro and in vivo and revealed that ribavirin is less effective for treating SFTSV infection than T-705 in IFNAR-/- mouse model [^19^](#_ENREF_19). Recently a report also demonstrated that T-705 protected the STAT2 knockout hamsters from SFTSV lethal infection [^20^](#_ENREF_20). These findings indicates that T-705 is a promising drug candidate for SFTSV infection and many other RNA viruses infections for which there are no approved therapies.

T-705 is a new anti-influenza drug approved for human use in Japan and is progressing through Phase 3 clinical trials in the United States in 2012 [^21^](#_ENREF_21). Until now, several studies reported the clinical efficacy of T-705 treatment in high lethal RNA virus infections. Three patients with Ebola virus infection recovered after receiving treatment of T-705 combined with monoclonal antibodies against Ebola virus (ZMAb for 2 patients and MIL77 for 1 patient) [^22-24^](#_ENREF_22). Two patients with Lassa virus infection recovered but had prolonged detectable virus RNA in blood and semen, after receiving treatment of T-705 combined with ribavirin [^25^](#_ENREF_25). One historically controlled single-arm clinical trial reported a case series of 39 patients with Ebola virus disease (EVD) who received T-705 treatment, indicating prolonged survival and markedly reduced viral load were associated with T-705 treatment [^26^](#_ENREF_26). Another clinical trial assessed the efficacy of T-705 in 99 adults and adolescents of EVD, showed minor role of the drug in treating EVD, although the patients with low viral loads indeed showed a lower mortality and a rapidly decreased viremia than the untreated value [^27^](#_ENREF_27).

There is no randomized clinical trial evaluating T-705 in treating high lethal RNA virus infections. The clinical use of T-705 has not been reported in treating SFTS, although its anti-SFTSV activity has been demonstrated in vitro and in animal models. Therefore, randomized clinical trials are warranted to test the efficacy and safety of treatment with T-705 in SFTS.

**2. Objectives**

This study is designed to evaluate the efficacy and safety of therapy with T-705 in SFTS patients. All subjects will be administered standard supportive measures. The study hypothesis is the standard supportive care plus T-705 will reduce the case fatality of SFTS.

**3. Study design**

This will be an investigator-initiated single-center, single-blinded, and randomized controlled trial evaluating the efficacy and safety of T-705 in treating SFTS. This study will be conducted at the People’s Liberation Army (PLA) 154 hospital, Xinyang, Henan province, China. Xinyang city located at the centre of the Dabie moutains which is the area of greatest SFTS endemicity in China, and the PLA 154 hospital received the majority of SFTS patients in this area. The eligible subjects who were agreed to participate in the study will be randomly assigned to receive oral T-705 in combination with supportive care or supportive care only. The researcher who allocated patients did not take charge of patients’ enrollment. Only patients were masked to the assigned drugs.

**4. Study population**

4.1 Subject selection

Subjects who seek treatment at the development of infectious diseases in the People’s Liberation Army 154 Hospital will be enrolled in this study.

4.2 Participants inclusion criteria

Clinical suspected SFTS patients with positive results for laboratory test of SFTSV infection were included into the study. Clinical suspected SFTS was defined as meeting all the following criteria: a) exposure history (field activities in SFTS-endemic areas or tick bites within 2 weeks); b) presentation of acute fever (temperature, of ≥37.5°C); c) laboratory abnormalities including thrombocytopenia (platelet count, <100 × 109 platelets/L) and/or leukopenia (leukocyte count, <4.0 × 109 leukocytes/L). Laboratory-confirmed SFTSV infection was defined as meeting 1 or more of the following criteria: a) detection of SFTSV RNA by a molecular method; b) isolation of SFTSV in cell culture; c) seroconversion or ≥4-fold increase of antibody titers between 2 serum samples collected at least 2 weeks apart.

4.3 Participants exclusion criteria

The exclusion criteria are as followed:

1) Patients aged <18 years;

2) Pregnant or lactational women;

3) Patients with chronic diseases (i.e. cancer, acquired immunodeficiency syndrome, diabetes, hepatitis, and pulmonary tuberculosis);

d) Patients infected with other vector-borne pathogens (e.g. Rickettsia sp., Borrelia sp., and Babesia sp.);

4) Patients complicated with underlying diseases (i.e. hematologic, renal, hepatic or autoimmune dysfunction);

5) Patients having a history of hypersensitivity to an antiviral nucleoside-analog drug targeting a viral RNA polymerase;

6) Patients currently using adrenocorticosteroids (except topical preparation) or immunosuppressive drugs;

7) Patients with contraindication for the use of T-705 (a history of gout and hyperuricemia).

4.4 Participants exit criteria

The exit criteria are as followed:

1) Patients forwardly withdrew from the study

2) Patients lost to the follow-up

3) Patients needed to withdraw due to other reasons

**5. Randomization and blinding**

This is a single-blind study. The eligible patients are randomly assigned by using a random number list generated by Microsoft Excel program (version 2013) in a 1:1 ratio, to receive the standard supportive care or the standard supportive care combined with T-705. The researcher who allocated patients does not take charge of patients’ enrollment. Only patients will be masked to the assigned drugs.

**6. Intervention**

Treatment will be generally initiated within 24 hours after randomization. All patients will receive standard supportive care, including supplement of electrolytes and dextrose, antipyretics, hepatoprotective, supplement of multivitamins, recombinant human granulocyte colony-stimulating factor, immunopotentiating agents, antiemetics, antidiarrheal, antibiotics, anticonvulsants, anxiolytics, corticosteroids, plasma transfusion, and platelet transfusion. The patients allocated to the T-705 treated group will additionally receive tablets of 1800 mg T-705 orally twice in the first day (3600 mg total), and tablets of 1000 mg twice on day 2. The administration of T-705 will last at least 5 days, or until to serum SFTSV RNA concentration is reduced below the low limit of quantification, or until to the patient is discharged from the hospital.

**7. Study schedule**

7.1 Screening and molecular detection

Subjects are firstly underwent physical examination and routine laboratory tests (hemogram and biochemical blood analysis) when being admitted to the hospital. Those who met the case definition for clinical suspected SFTS have their serum samples collected. The serum samples are subjected to detection of SFTSV RNA by reverse transcriptase polymerase chain reaction. Suspected SFTS patients with positive SFTSV RNA detection are defined as laboratory-confirmed SFTS patients. Clinicians will introduce this trial to these laboratory-confirmed SFTS patients. All these laboratory-confirmed SFTS patients are further interviewed by health-care clinicians using a standardized questionnaire, including previous history of diseases and medicine history, to assess their eligibility into the study. Written consent informs should be signed before the questionnaire.

7.2 Monitoring of clinical manifestations and laboratory abnormalities

After the subjects enrolled into the study, their clinical manifestations are recorded daily on a structured case report form by doctors and nurses on duty until their signs/symptoms resolved or their viremia turned negative or they were discharged from the hospital. During the patients’ hospitalization, routine laboratory tests, including hemogram and biochemical blood analysis, are prescribed at least every other day.

7.3 Monitoring of viremia

During the patients’ hospitalization, blood samples are collected for the assessment of serum SFTSV RNA concentrations with the use of reverse transcriptase polymerase chain reaction.

7.4 Follow up

When the patients are discharged from the hospital, their outcomes are retrieved from medical records. For the patients who discontinue therapy or are discharged from the hospital because of adverse clinical progression or for economic reasons, the clinicians will perform a follow-up by phone call or home visit to determine their final outcome.

**8. Adverse effects**

Severe adverse effects related to the administration of T-705 included skin allergy, pneumonitis, jaundice, toxic epidermal necrolysis, mucocutaneocular syndrome, acute renal failure, hemorrhagic colitis, and neurologic symptoms. Other adverse effects include: gastrointestinal symptoms (i.e. nausea, vomiting, abdominal pain, and diarrhea) and laboratory abnormalities (i.e. leucocytopenia, thrombocytopenia, elevated liver enzyme level, and elevated serum uric acid level). During the patients’ hospitalization, adverse effects related to the administration of T-705 are recorded daily on a structured case report form by doctors and nurses. If presenting severe adverse events during treatment, the subjects should be intensively monitored. Based on the deterioration that may be related to the administration of T-705, the clinicians will stop the therapy with the study drug. All survived patients will be asked to return for clinical assessment when one month after discharge from the hospital to evaluate the long-lasting effect, including hemogram and biochemical blood analysis, and serum viremia.

**9. Outcomes**

9.1 Primary outcome

The primary outcome is case fatality. The fatal outcome was firstly retrieved from medical records, and further verified by performing a follow-up visit one week after discharge.

9.2 Secondary outcomes

The secondary outcomes include: a) the time needed for viral clearance and dynamic changes of viral load during hospitalization; b) the development of severe complications such as hemorrhagic signs, neurological symptoms, and dyspnea; c) the dynamic changes of laboratory parameters such as aspartate aminotransferase, lactate dehydrogenase, creatine kinase, platelet count, neutrophil percentage, and lymphocyte percentage.

**10. Data analysis**

10.1 Data entry and management

Data entry is completed by 2 individuals who do not participate in the randomization. All data will be cross-checked once a week. The data manager will conduct monthly comprehensive data checks for identifying systematic errors or problems. If any problem is found, the supervisor should be informed in time, and the researcher should be required toresolve the problem. The raw experimental data is exported and checked using the device specialized software. All data will be regularly stored locally.

10.2 Sample size calculation

Based on our previous observational study, the case fatality rate of laboratory-confirmed SFTS patients from May to August is set is about 17%. This study aims to test a relative difference of 85% in case fatality rate between the T-705 treatment group and the control group. The minimal sample size is 70 for each group with statistical power of 85% at a significance level of 0.05.

10.3 Data analysis plan

The primary analysis objectives of this study are:

1) To determine the efficacy of treatment with T-705 in subjects with SFTSV infection, as measured by the case fatality rate;

2) To evaluate the safety of T-705 treatment in subjects with SFTSV infection.

The secondary analysis objectives of this study are:

1) To evaluate the effect on viral clearance of T-705 treatment in subjects with SFTSV infection;

2) To evaluate the effect on severe complications development of T-705 treatment in subjects with SFTSV infection;

3) To evaluate the effect on kinetics of key laboratory parameters of T-705 treatment in subjects with SFTSV infection.

For baseline characteristics, the data on demographic (i.e. age and gender), vital signs (i.e. body temperature, pulse, and blood pressure), clinical features (i.e. delay from symptom onset to admission and signs/symptoms occurred before admission), laboratory test results (blood hemogram and biochemical parameters), and SFTSV viral load, will be compared between the T-705 treated group and the control group. The differences of baseline data between two groups are assessed by the two-sample t test or the nonparametric Mann-Whitney test for continuous variables, and by the Chi-square test or the Fisher’s exact test for categorical variables. The choice of the test depends on the distributional characteristics of the data.

The analysis of primary outcome (case fatality rate) will use a time-to-event approach to examine differences between the T-705 treated group and the control group. Kaplan-Meier survival curve is plotted and the log-rank test is used to compare treatment arms. Based on our previous findings that age and delay from symptom onset to hospital admission are associated with fatal outcome of SFTS, these two factors, together with gender, will therefore be adjusted by performing Cox proportional hazards model to estimate hazard ratios and 95% confidence intervals for treatment. The analysis for safety is to compare the proportions of subjects with adverse events between the T-705 treated group and the control group.

For analysis of secondary outcomes, the two-sample t test is applied to compare the time needed for viral clearance between the two groups; the Chi-square test or the Fisher’s exact test is applied to compare the frequency of severe complications that are developed during treatment between the two groups. Generalized estimating equation model will be used to analysis the data on daily measurements of viral loads and laboratory parameters.

Further post-hoc subgroup analyses will be performed for the primary outcome with regard to baseline viral load, delay from symptom onset to hospital admission, age, and sex. Considering that the baseline viral load affects the treatment benefit, post-hoc subgroup analyses will also be performed for the secondary outcomes.

**11. Ethics**

Before the start of the study, the study protocol has been approved by the Ethics Committee of People’s Liberation Army 154th Hospital, and the approved number of ethic committee is 154YY-LL-2018-02.

**Figure S1. Flowchart of the next-generation sequencing of SFTSV genome in 4 passages of cell culture supernatant and intracellular SFTSV genome.**

For experiments involving serial passage of virus in the presence of T-705, passage 1 cells were infected with SFTSV and treated with T-705. In subsequent passages, 200 μl supernatant of the previous passage (1/5 of total virus) were added to a new ls (step 1). A total of 4 passages were performed, and both cells and supernatant were collected. SFTSV RNAs were extracted from both cell and supernatant (step 2). For NGS analysis, samples were prepared using NEBNext Ultra™ RNA Library Prep Kit for Illumina (NEB), and then sequenced on an Illumina MiSeq generating a 150-bp paired-end reads (step 3).

**
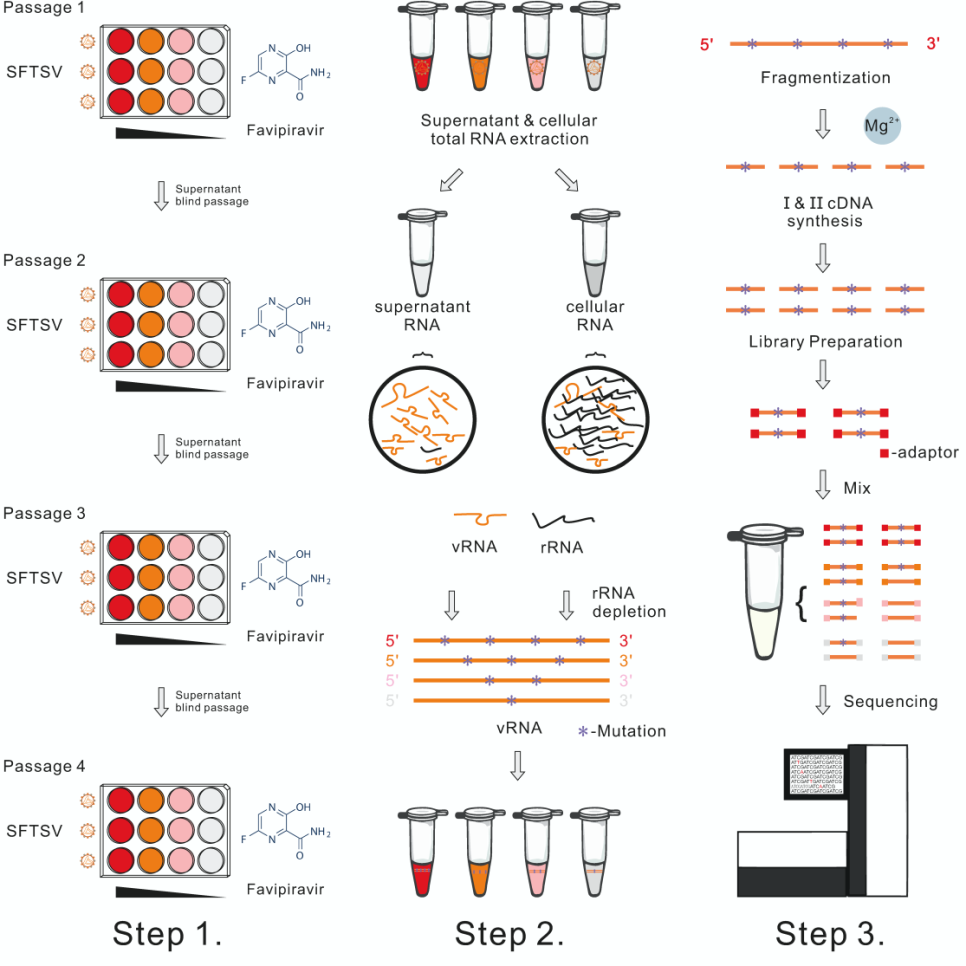
**

**Figure S2. Viral load and mutation analysis of intracellular SFTSV from cells in 4 passages treated with different concentrations of T-705.**

The NGS and mutation analysis of intracellular SFTSV genome collected from T-705 treated/no T-705 treated cells. Total mutation rates (**a**), ratios of transitions/transversions (**c**), transition mutation rates (**d**), and percentages of different transition mutations (**e**) were calculated. Relative intracellular level of viral genome was measured (**b**).

**
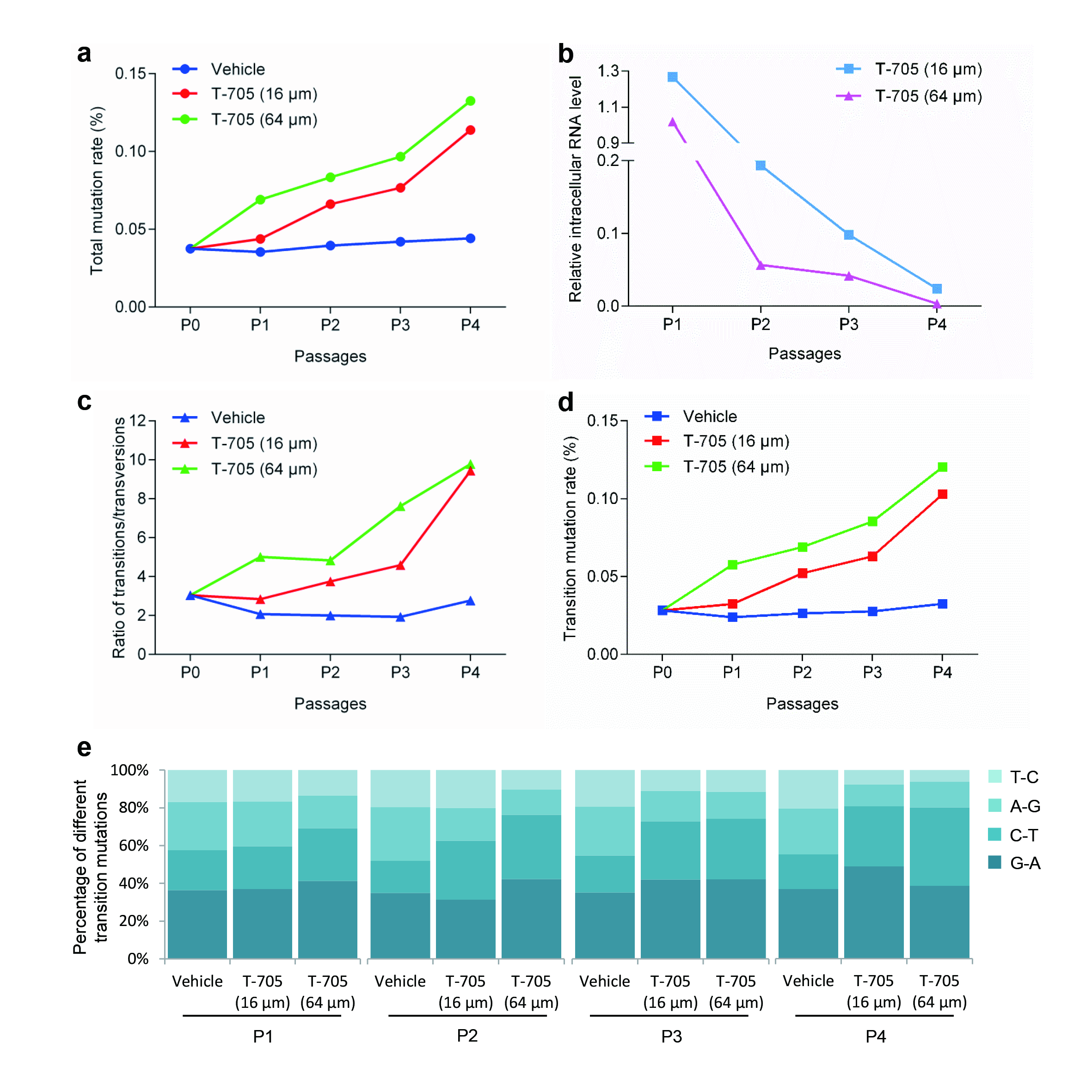
**

**Figure S3. Percent of SNV frequency across the SFTSV genome displays increased viral sequence diversity.**

For experiments involving serial passage of virus in the presence of T-705, passage 1 cells were infected with SFTSV and treated with T-705. In subsequent passages, 200 μl supernatant of the previous passage (1/5 of total virus) were added to a new monolayer of Vero cells. A total of 4 passages were performed, and cells were collected and SFTSV RNAs were extracted for NGS analysis. SNV frequency was calculated, and percent of SNV frequency (y axis) across the SFTSV genome (x axis) was displayed, with left to right indicating L, M, and S segments. (**a**) SNVs frequencies for cells and supernatants in P1-P3; (**b**) SNVs frequencies for cells and supernatants in P4.

**
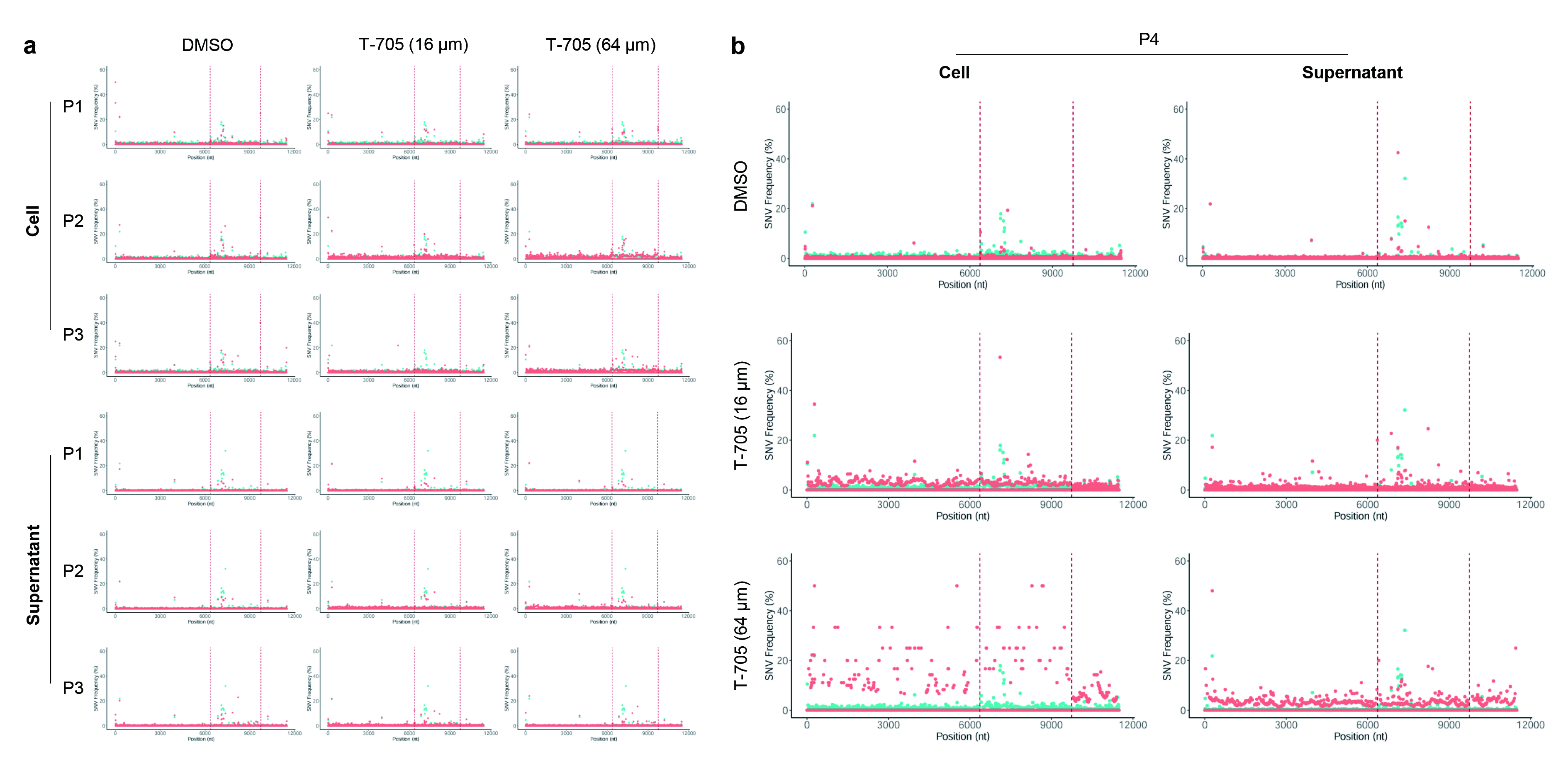
**

**Figure S4. Flowchart of the next-generation sequencing of SFTSV genome in cell culture supernatant and intracellular SFTSV genome collected from cells infected with different MOIs of SFTSV.**

Cells were infected with SFTSV at MOIs of 1, 10 and 30, and treated with different concentrations of T-705 (step 1). At 24 hours p.i., SFTSV RNAs were extracted from both cell and supernatant (step 2). For NGS analysis, samples were prepared using NEBNext Ultra™ RNA Library Prep Kit for Illumina (NEB), and then sequenced on an Illumina MiSeq generating a 150-bp paired-end reads (step 3).

**
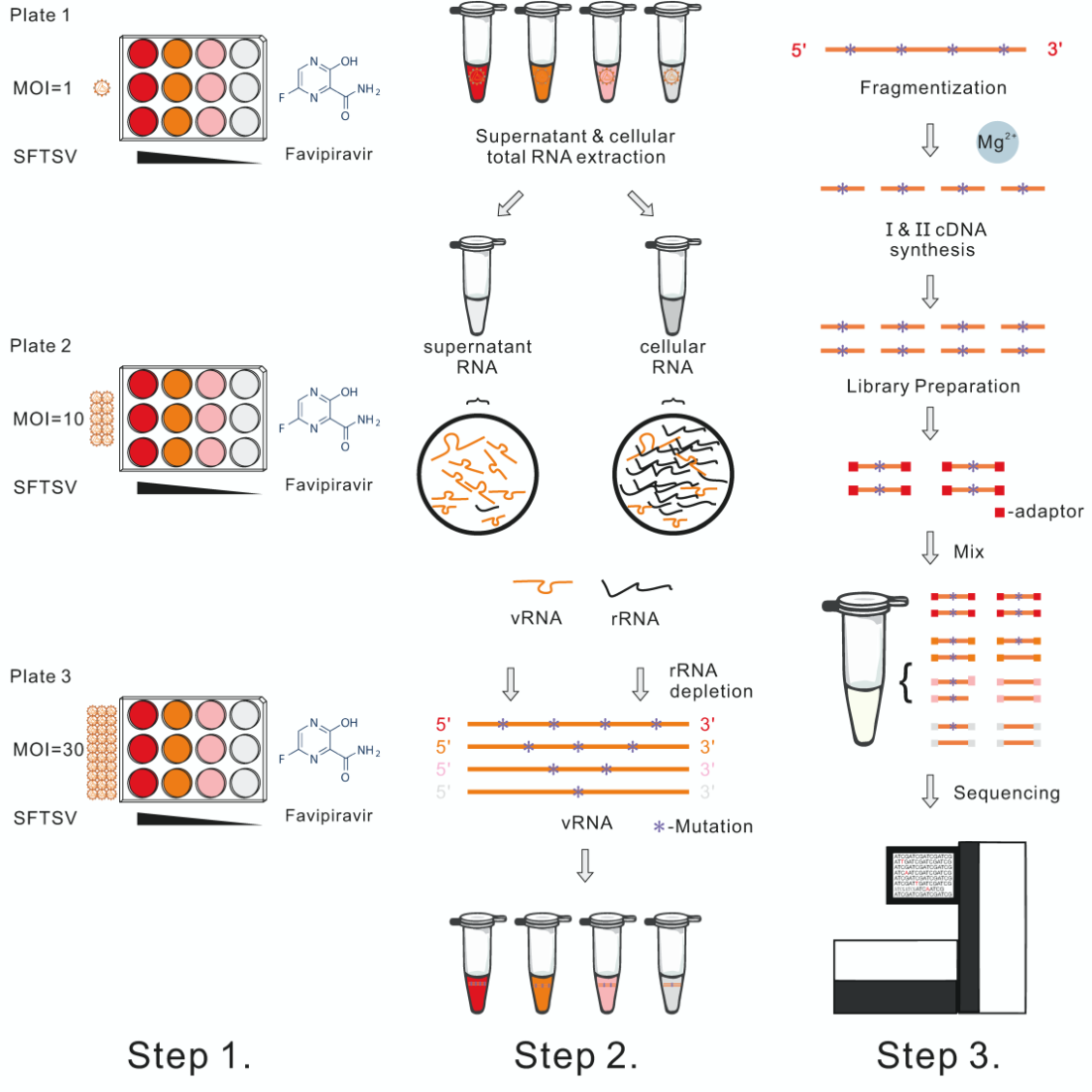
**

**Figure S5. Viral load and mutation analysis of intracellular SFTSV in cells treated with different concentrations of T-705.**

Vero cells were infected with SFTSV strain HNXY2017-66 at MOIs of 1, 10, and 30, and treated with vehicle or different concentrations of T-705. And at 24 hours post infection, both supernatant and cells were collected. SFTSV genome in cells was extracted and subjected to NGS analysis. Total mutation rates (**a**), ratios of transitions/transversions (**c**), transition mutation rates (**d**), and percentages of different transition mutations (**e**) were calculated. Relative intracellular level of viral genome was measured (**b**).

**
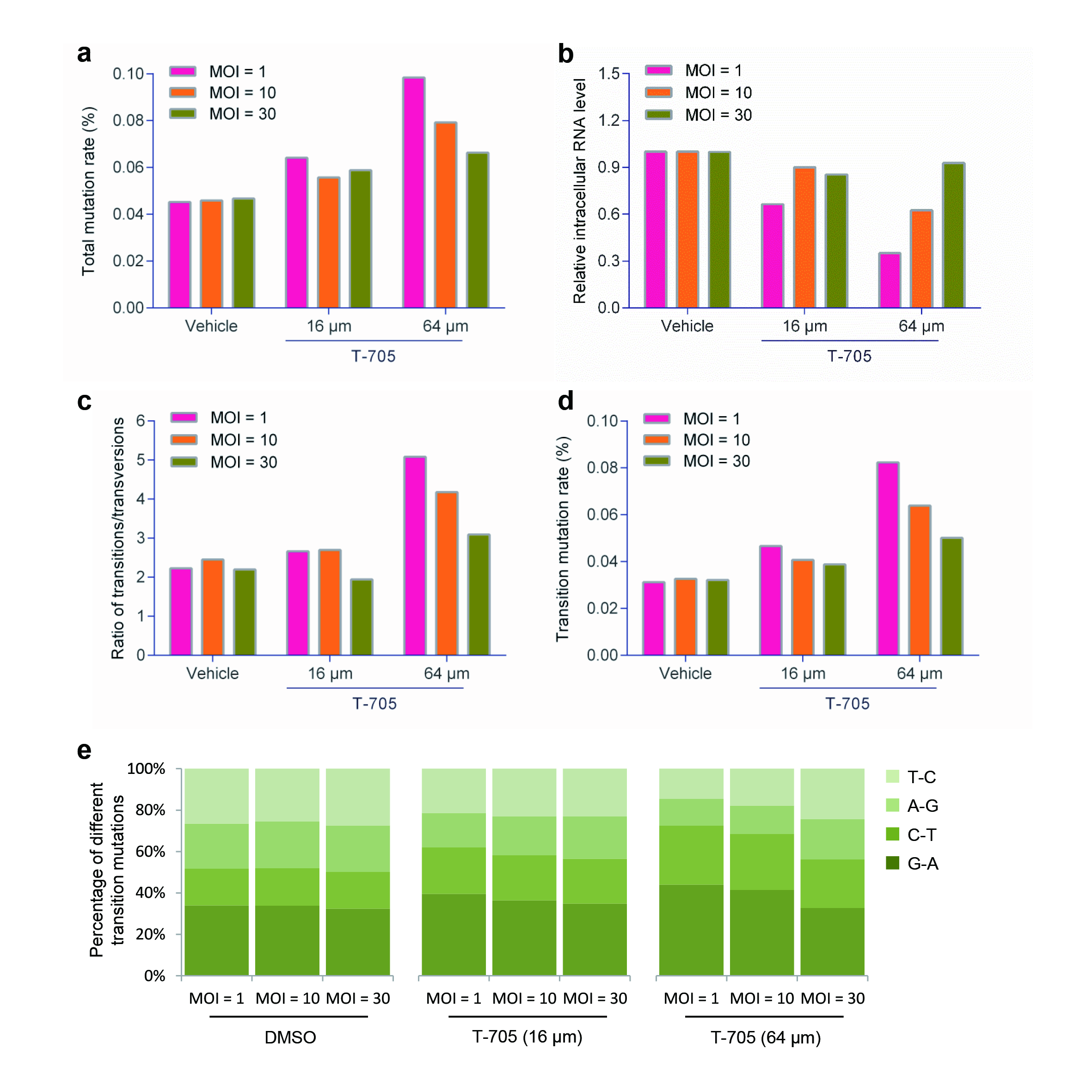
**

**Figure S6. Patterns of transition and transversion mutations in serum samples of mice by T-705 treatment over time.**

At days 2, 3, 4 post SFTSV infection, mice were scarified, and spleen and serum samples were collected. SFTSV genome was extracted from the serum and subjected to NGS and mutation analysis. (**a-c**) Patterns of transition mutations; (**d-f**) Patterns of transversion mutations.

**
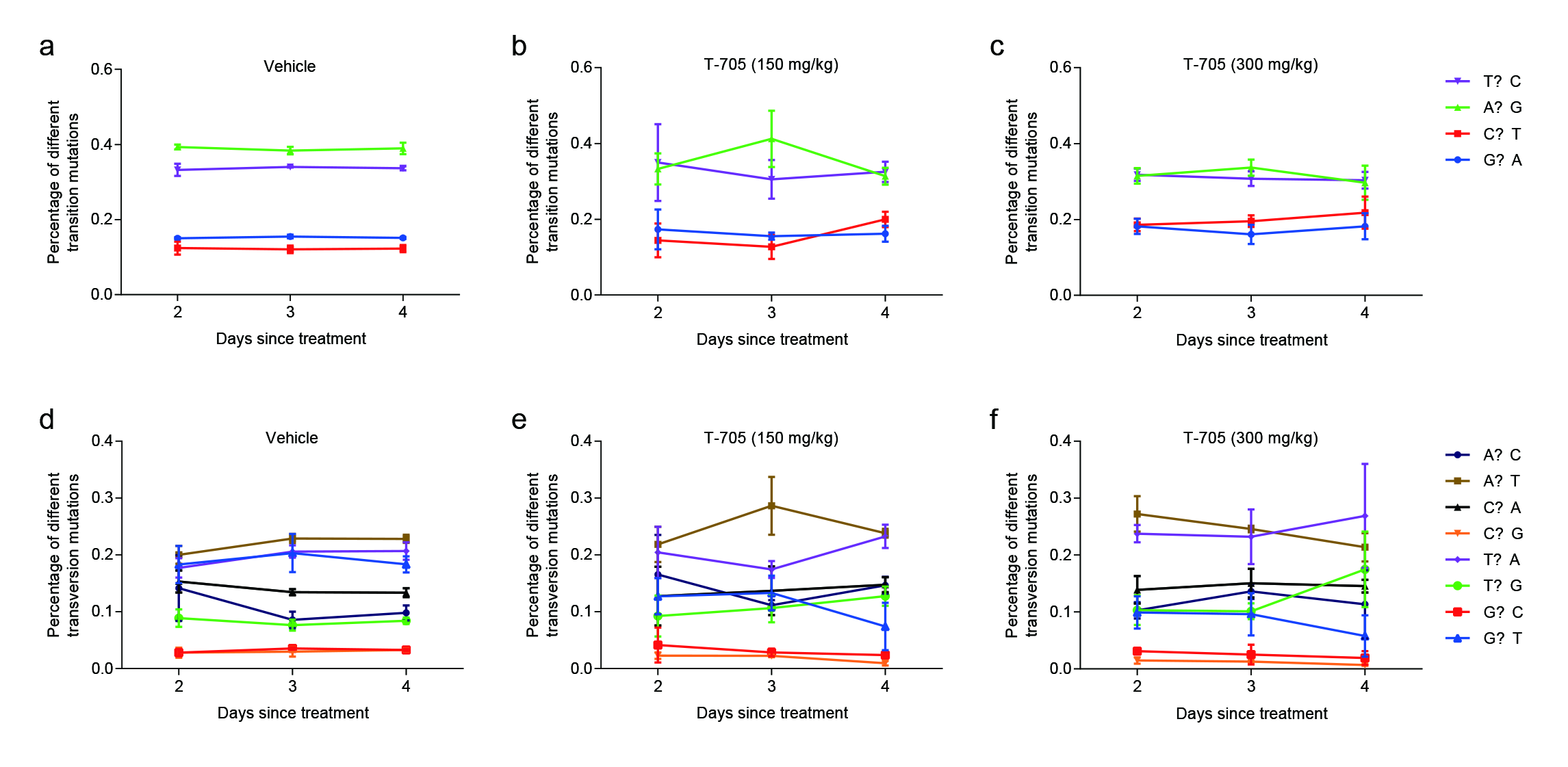
**

**Figure S7. The NGS and mutation analysis of SFTSV genome in spleen samples collected from IFNAR^-/-^ C57BL/6 mouse treated with or without T-705.**

Three IFNAR^-/-^ C57BL/6 mice from each group were sacrificed on day 2, 3, and 4 post infection, respectively. Serum and spleen samples were collected, and RNA was extract for NGS analysis. Copy number of SFTSV genome in spleen (**a)**, the total mutation rates (**b**), transition mutation rates (**c**), transversion mutation rates (**d**), and ratios of transitions/transversions (**e**) were also calculated.

**
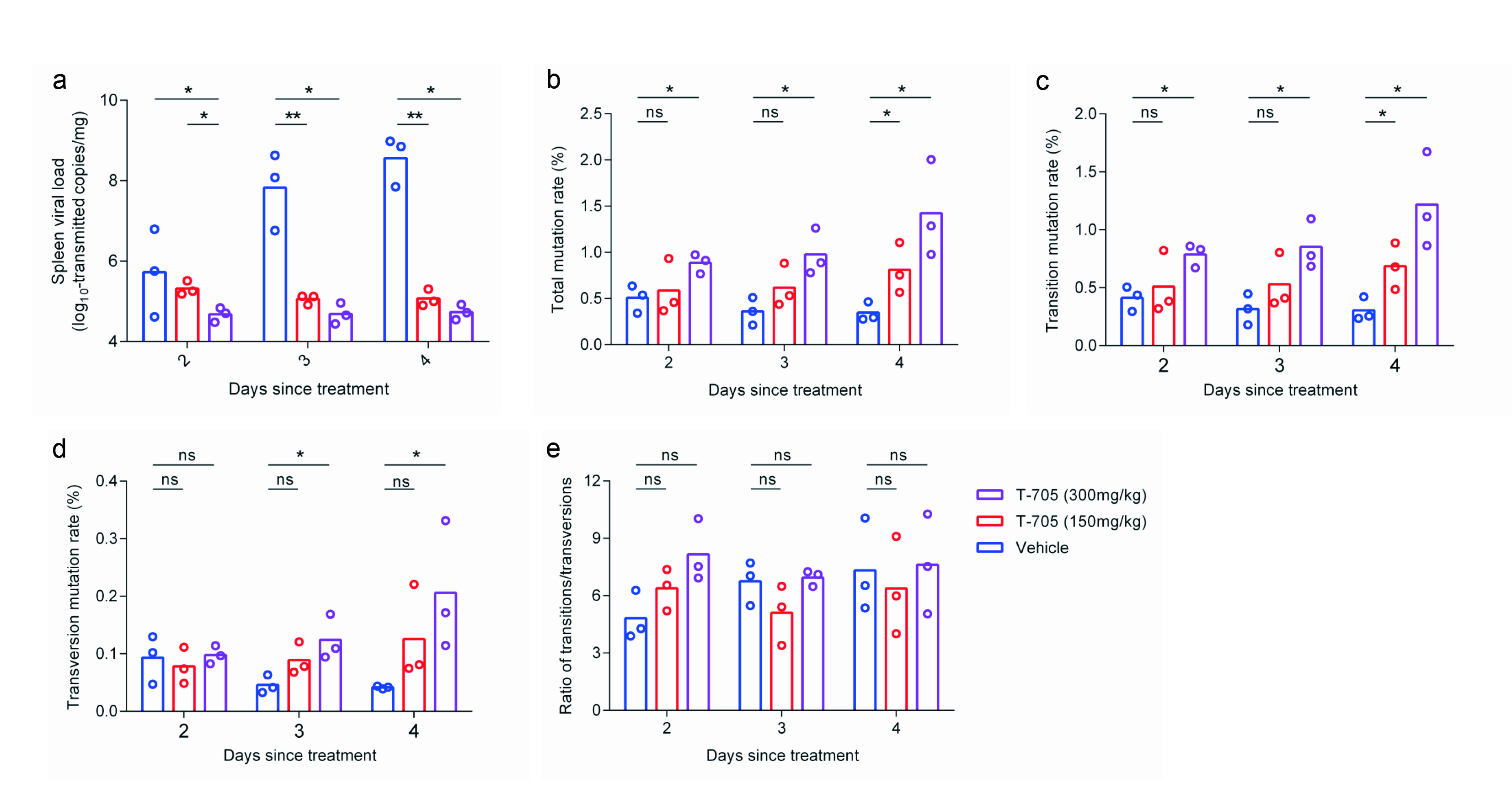
**

**Figure S8. Baseline viral loads of the SFTS patients.**

(**a**)The mean (standard deviation) values of RT-PCR cycle-thresholds; (**b**) The number of the patients who had the baseline value of RT-PCR cycle-threshold <26 or ≥26.

**
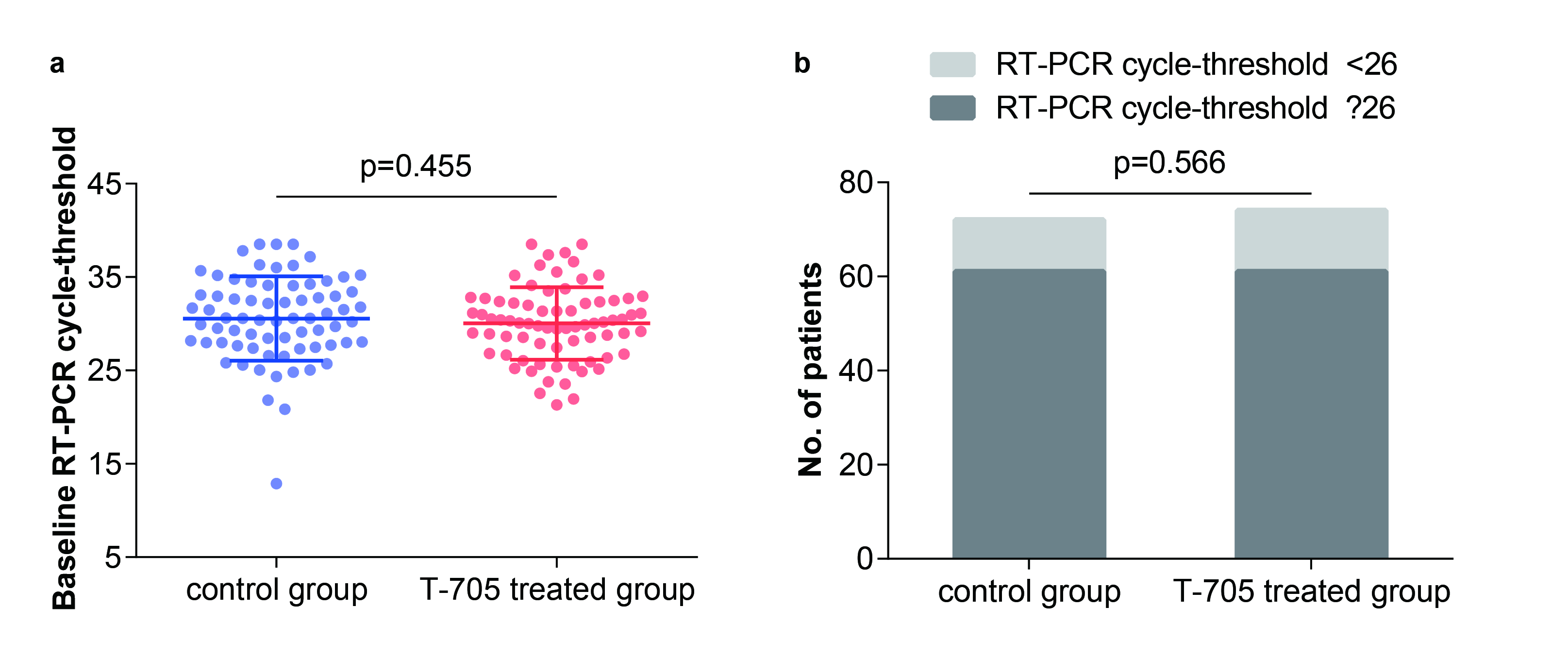
**

**Figure S9. Kaplan-Meier curves for the treatment effect on probability of survival stratified by age, gender and hospital admission delay.**

Kaplan-Meier curves with 95% confidence bands are shown by treatment arm based on the delay from symptom onset to hospital admission (<6 days, N=99, Panel **a**; ≥6 days, N=46, Panel **b**), the age (<60 years, N=47, Panel **c**; ≥60 years, N=98, Panel **d**), and the gender (male, N=57, Panel **e**; female, N=88, Panel **f**).


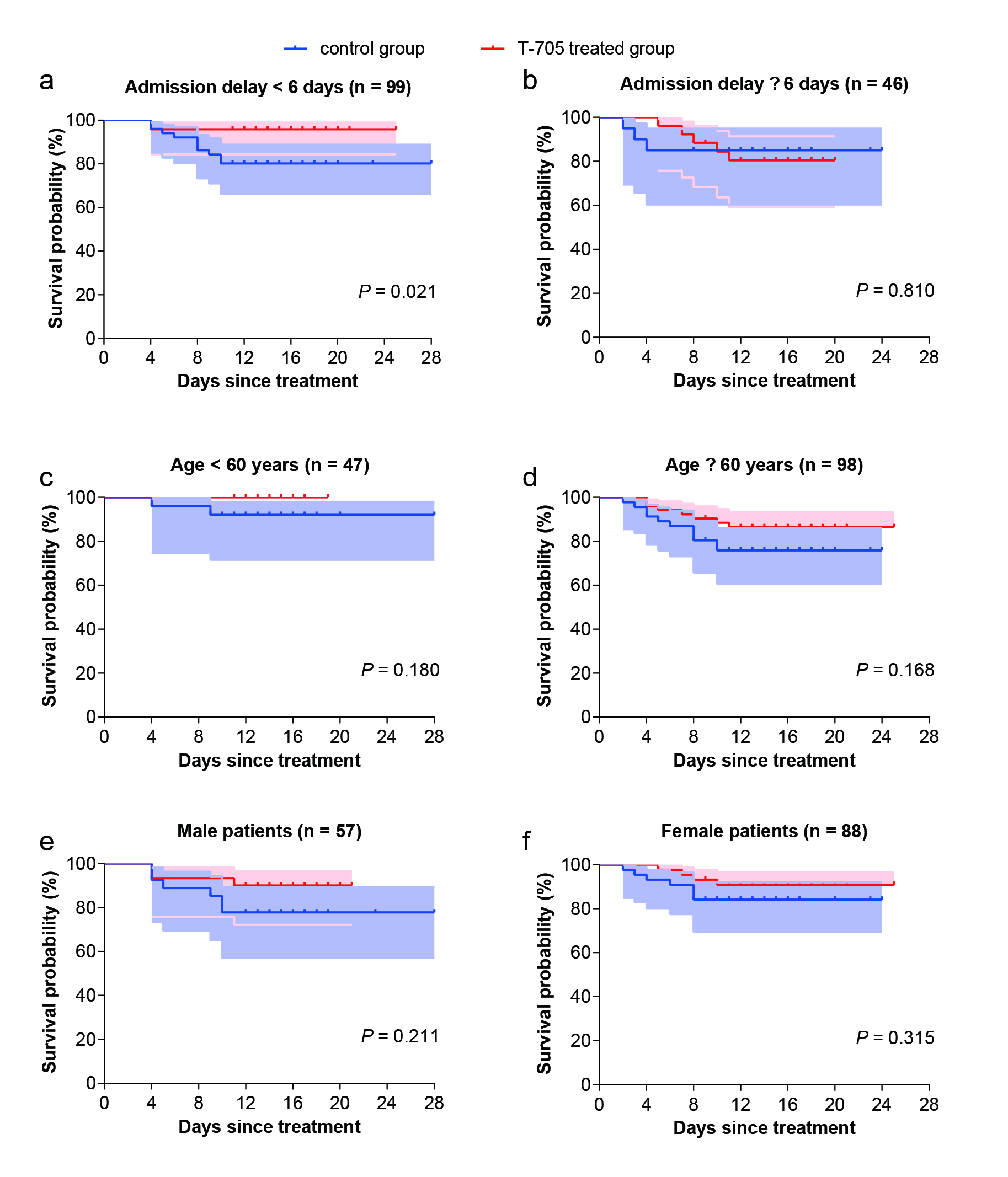


**Figure S10. The kinetics of the SFTSV Viral Load**.

The mean (95% confidence intervals) values of RT-PCR cycle threshold over time are shown for all patients (**a**) and for patients (**b**) with low-baseline viral loads. The numbers of patients who contributed to the at-risk population at each time point are shown under the x-axis.


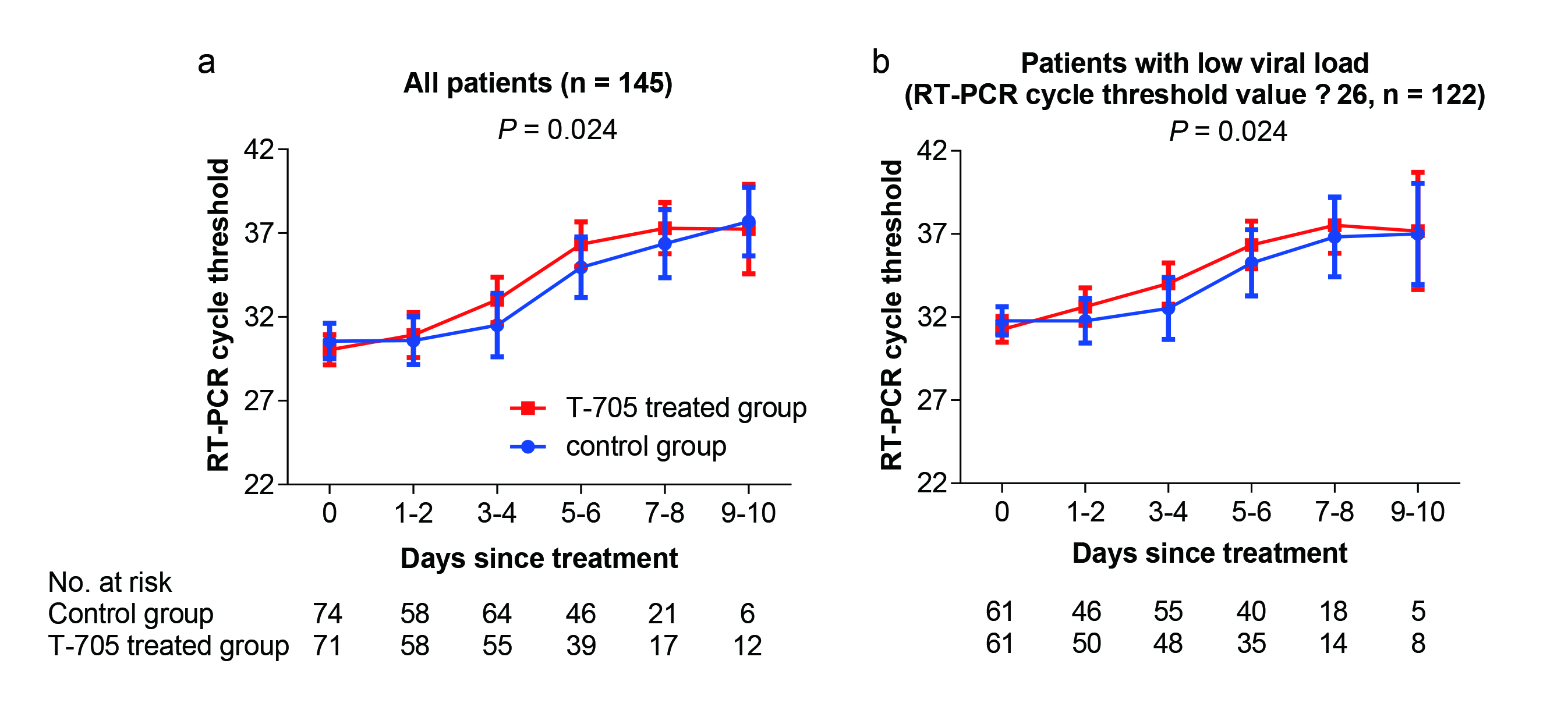


**Figure S11. The kinetics of key laboratory parameters in the patients.**

The median (quartile range) values of platelet count, aspartate aminotransferase, aspartate aminotransferase, lactate dehydrogenase, and creatine kinase are shown over time for all the patients (**a**, **c**, **e**, and **g**), and the patients with low baseline viral loads (RT-PCR cycle-threshold value ≥26) (**b**, **d**, **f**, and **h**). The numbers of patients who contributed to the at-risk population at each time point are shown under the x-axis. The difference of these severe complications was analyzed over time (the curves) by using the generalized estimating equation model.


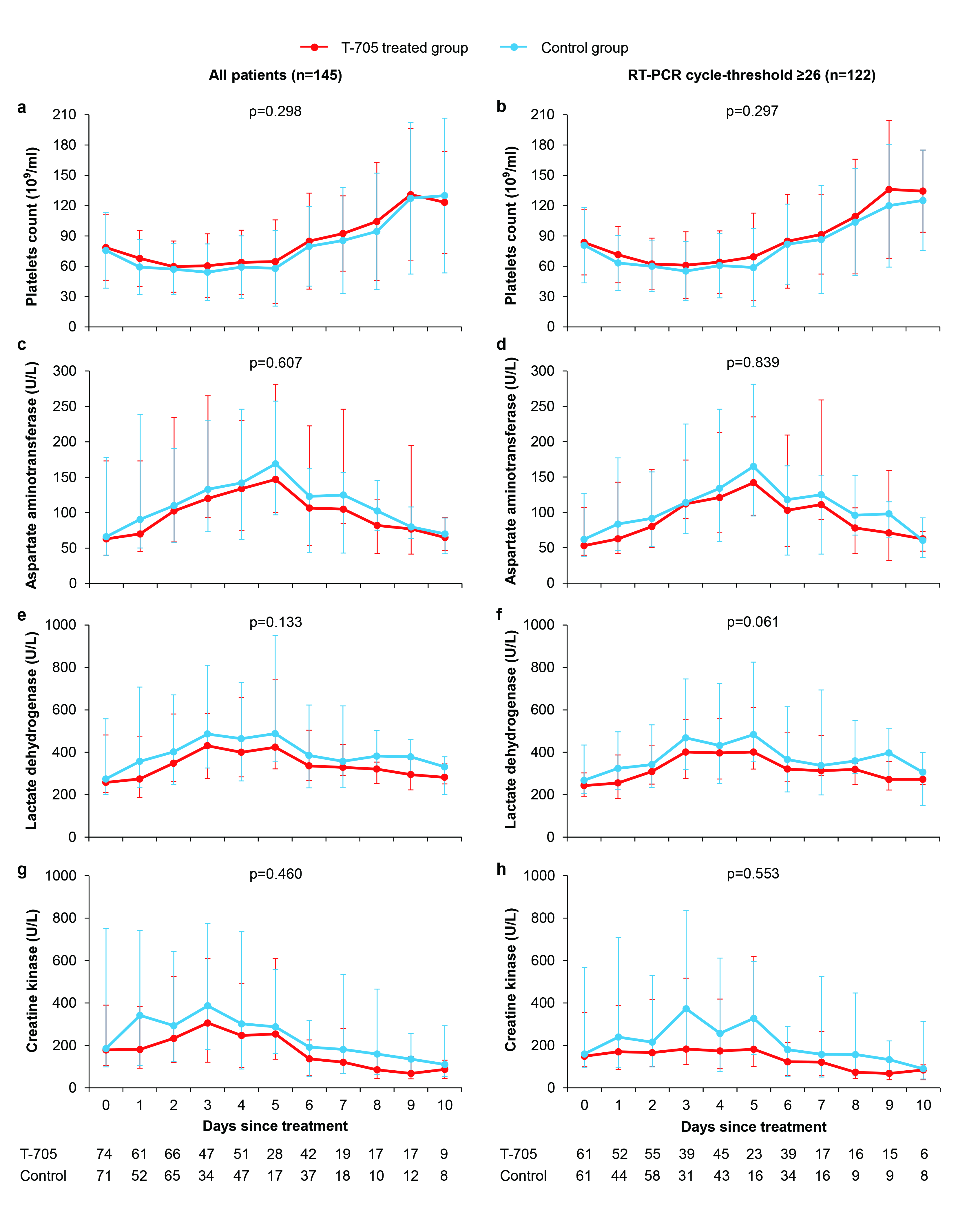


**Figure S12. The kinetics of uric acid in the patients.**

The median (quartile range) values of uric acid are shown over time for all the patients (**a**), the male patients (**b**), and the female patients (**c**). The dotted line indicates the upper limit of normal range.


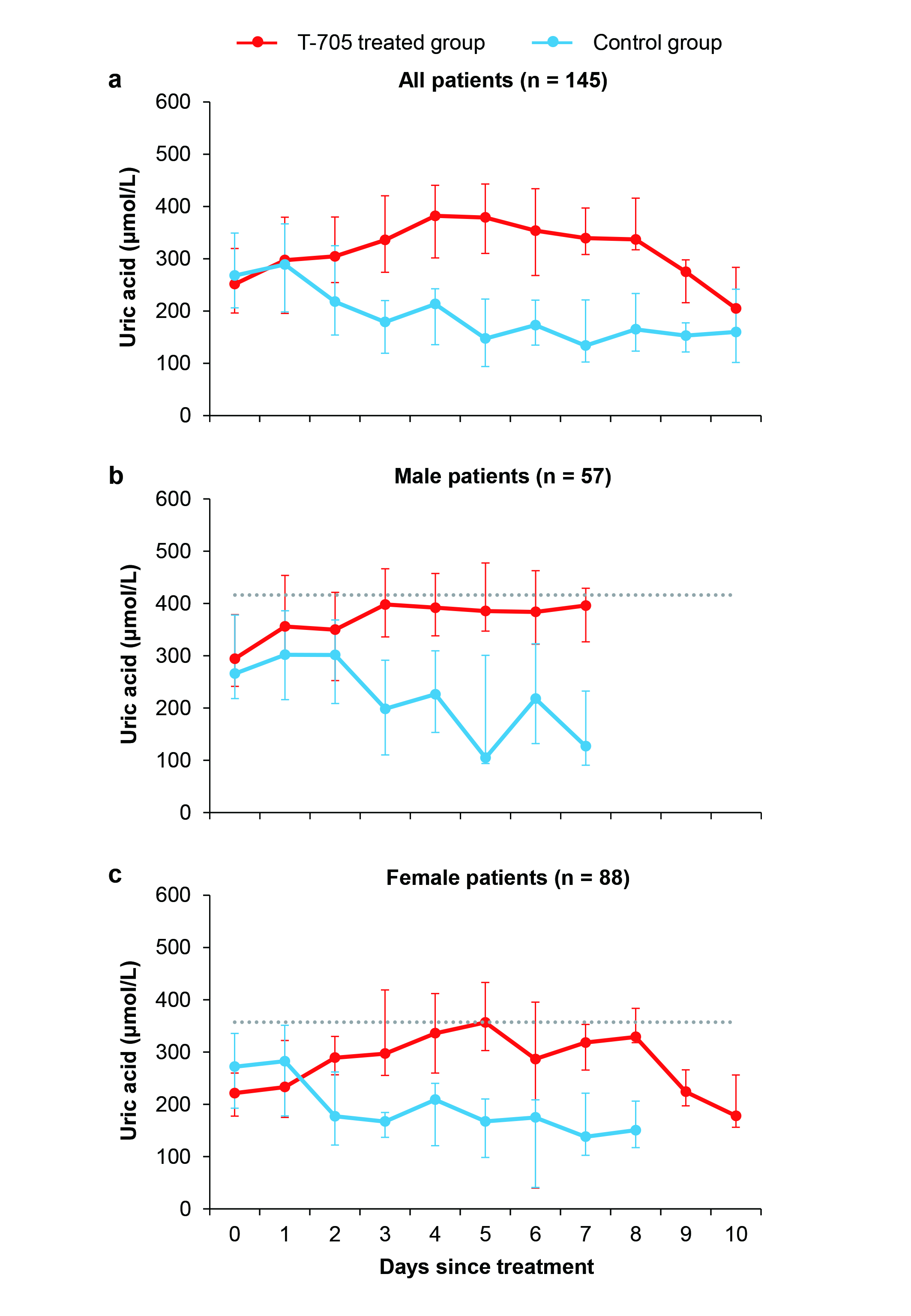


**Figure S13. The ratio of transitions/transversions in the patients treated with or without T-705.**

A total of 40 samples were obtained from 12 patients receiving T-705 treatment (4 fatal and 8 survival) and 34 samples were obtained from 11 controls (5 fatal and 6 survival). The mean ratios of transitions/transversions are shown over time for the two groups.


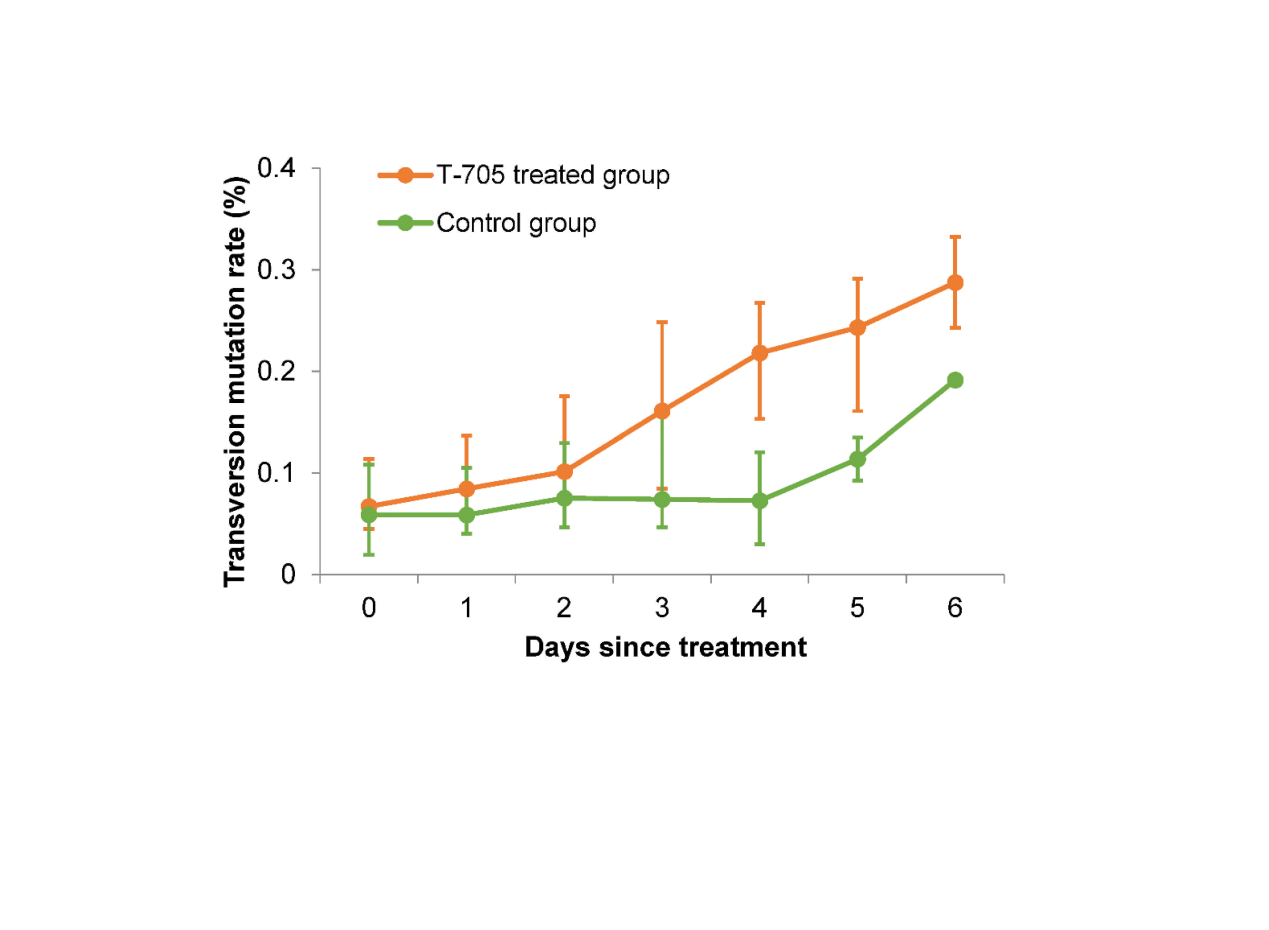


**Figure S14. The kinetics of the viral load in SFTS patients.**

A total of 40 samples were obtained from 12 patients receiving T-705 treatment (4 fatal and 8 survival) and 34 samples were obtained from 11 controls (5 fatal and 6 survival). The daily value of RT-PCR cycle threshold was shown over time for each patient in the T-705 treated group (**a**) and the control group (**b**).


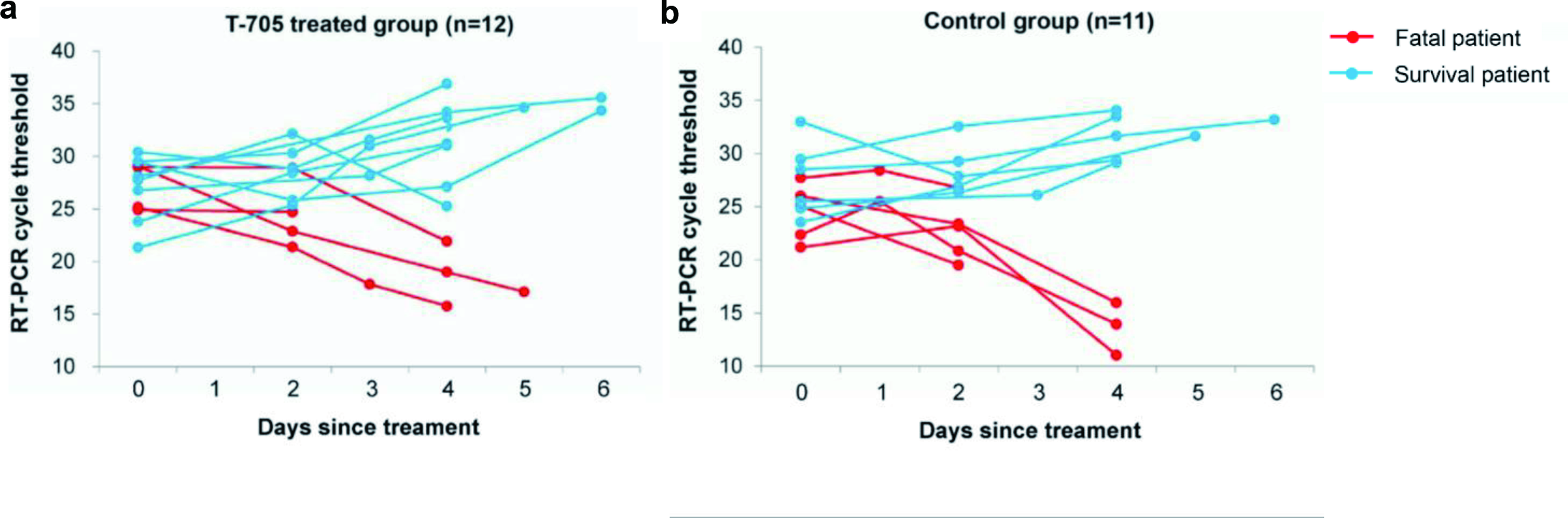


**Figure S15.** **Patterns of transition and transversion mutations in serum samples of SFTS patients by T-705 treatment over time.**

Serum samples were collected at days 2, 3, 4 after T-705 treatment, and RNA was extracted and subjected to NGS and mutation analysis. (**a, b**) Patterns of transition mutations; (**c, d**) Patterns of transversion mutations.

**
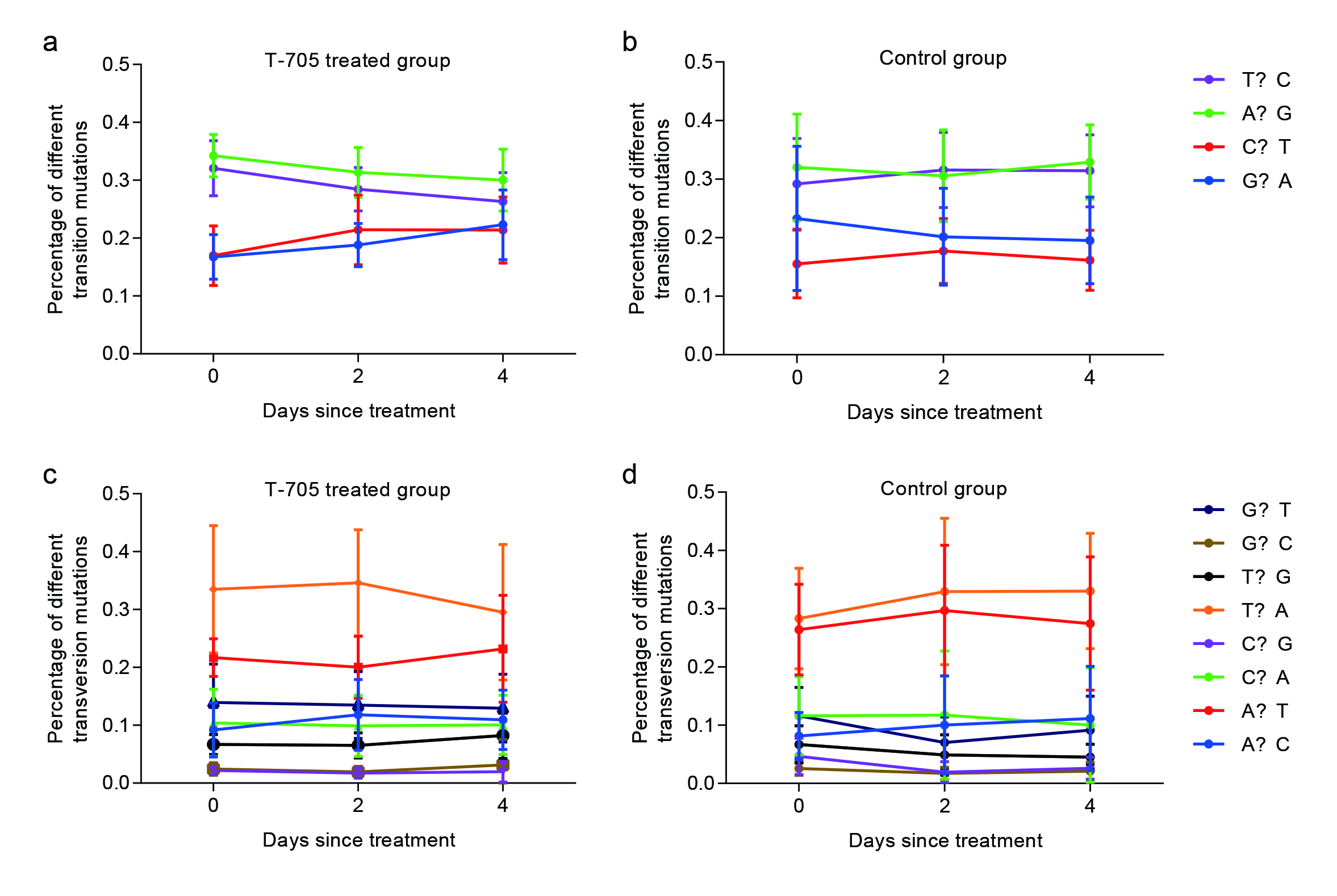
**

**Figure S16. Serum cytokines in the patients treated with or without T-705.**

Serial serum samples were collected from the SFTS patients who were included the clinical trial, and were tested for levels of several cytokines by using Luminex system. A total of 31 samples obtained from 9 patients receiving T-705 treatment (4 fatal and 5 survival) and 31 samples obtained from 10 controls (4 fatal and 6 survival) were included for analysis. The serum levels of IL-10, IL-6, IFN-γ, TNF-α, and GM-CSF are shown over time for the patients in the T-705 treated group (**a**, **c**, **e**, **g**, **i**) and the patients in the control group (**b**, **d**, **f**, **h**, **j**).

**
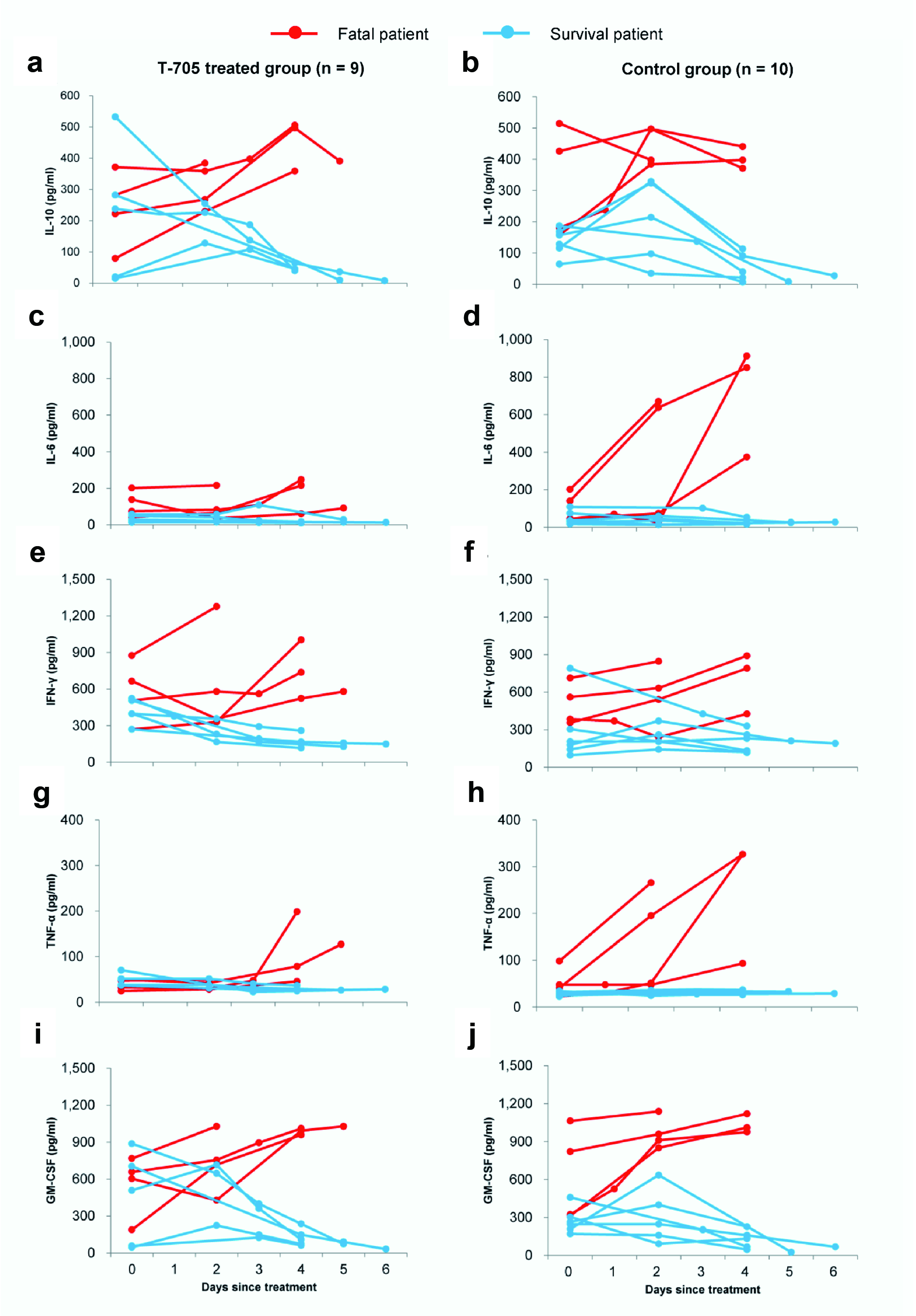
**

**Table S1. Baseline laboratory test results of the SFTS patients in the T-705 treated group and the control group.**

| **Variables**^*^ | **T-705 treated group (n=74)**^†^ | **Control group (n=71)**^†^ | ***P* value** |
| --- | --- | --- | --- |
| PLT — 10^9^/L | 78 (49.5-98) | 70 (49-90) | 0.358 |
| WBC — 10^9^/L | 2.1 (1.6-2.9) | 2.4 (1.8-3.3) | 0.062 |
| NEU percentage — % | 68.8 (57.2-76.0) | 68.5 (56.4-76.9) | 0.913 |
| LYM percentage — % | 25.2 (18.8-35.8) | 24.7 (18.0-33.9) | 0.747 |
| RBC — 10^12^/L | 4.2±0.5 | 4.2±0.5 | 0.688 |
| HGB — g/L | 128±15 | 126±17 | 0.443 |
| AST — U/L | 63 (40-173) | 66 (40-178) | 0.652 |
| ALT — U/L | 30 (21-61) | 38 (21-72) | 0.457 |
| CK — U/L | 179 (107-390) | 185 (99-751) | 0.401 |
| LDH — U/L | 258 (211-482) | 274 (210-558) | 0.340 |
| ALB — g/L | 39.3±4.8 | 38.0±4.2 | 0.093 |
| TBIL — μmol/L | 8.5 (6.6-11.4) | 9.8 (7.0-13.5) | 0.083 |
| BUN — mmol/L | 6.7 (5.4-7.8) | 6.2 (4.4-8.0) | 0.359 |
| UA — μmol/L | 251.5 (196.5-320.0) | 268.0 (206.0-349.0) | 0.456 |
| CREA — μmol/L | 78 (67-92) | 84 (66-105) | 0.224 |
| PT — S | 11.2±2.1 | 11.1±1.2 | 0.546 |
| APTT — S | 36.4±8.6 | 37.2±12.0 | 0.652 |
| TT — S | 21.9±8.6 | 21.0±4.8 | 0.539 |
| FIB — g/L | 2.95±0.71 | 2.76±0.80 | 0.168 |
| INR | 0.99±0.20 | 0.96±0.11 | 0.410 |

^*^Continuous variables are represented as mean (SD) or median (interquartile range).

^†^A total of 129 patients had available data, including 68 in the T-705 treated group and 61 in the control group.

PLT, platelet; WBC, white blood cell; NEU, neutrophil; LYM, lymphocyte; RBC, red blood cell; HGB, hemoglobin; AST, aminotransferase; ALT, aspartate aminotransferase; CK, creatine kinase; LDH, lactate dehydrogenase; ALB, albumin; TBIL, total bilirubin; BUN, blood urea nitrogen; UA, uric acid; CREA, creatinine; PT, prothrombin time; APTT, activated partial thromboplastin time; TT, thrombin time; FIB, fibrinogen; INR, international normalized ratio. Normal ranges for each variable are 100-300 10^9^/L for PLT, 4-10 10^9^/L for WBC, 50-70% for NEU percentage, 20-40% for LYM percentage, 3.5-5.5 10^12^/L for RBC, 110-170 g/L for HGB, 0-40 U/L for AST, 0-40 U/L for ALT, 25-200 U/L for CK, 109-245 U/L for LDH, 35-55 g/L for ALB, 5.11-17.10 μmol/L for TBIL, 2.86-8.20 mmol/L for BUN, 210-430 μmol/L for UA, 40-79 μmol/L for CREA, 9.6-14.0 S for 23.0-35.0 S for APTT, PT, 14.0-21.0 S for TT, 2.0-4.0 g/L for FIB, and 0.8-1.2 for INR.

**Table S2. The supportive treatment regiments during hospitalization of the SFTS patients in the T-705 treated group and the control group.**

| **Variables** | **T-705 treated (n=74)**^*^ | **Control group (n=71)**^*^ | ***P* value** |
| --- | --- | --- | --- |
| Sodium chloride | 74 (100) | 71 (100) | 1.000 |
| Dextrose | 74 (100) | 71 (100) | 1.000 |
| Potassium chloride | 66 (89.2) | 60 (84.5) | 0.404 |
| Antipyretic | 63 (85.1) | 53 (74.6) | 0.115 |
| Hepatoprotective | 62 (83.8) | 59 (83.1) | 0.912 |
| Multivitamin | 60 (81.1) | 53 (74.6) | 0.350 |
| Recombinant human granulocyte colony-stimulating factor | 64 (86.5) | 53 (74.6) | 0.092 |
| Immunopotentiating agents | 45 (60.8) | 49 (69.0) | 0.301 |
| Antiemetics | 38 (51.4) | 39 (54.9) | 0.666 |
| Antibiotics | 33 (44.6) | 34 (47.9) | 0.691 |
| Plasma transfusion | 37 (50.0) | 34 (47.9) | 0.799 |
| Corticosteroids | 19 (25.7) | 23 (32.4) | 0.373 |
| Creatine phosphate sodium | 18 (24.3) | 23 (32.4) | 0.281 |
| Antidiarrheal | 25 (33.8) | 15 (21.1) | 0.088 |
| Respiratory ventilation improvement | 23 (31.1) | 17 (23.9) | 0.336 |
| Platelets transfusion | 16 (21.6) | 15(21.1) | 0.942 |
| Albumin | 15 (20.3) | 12 (16.9) | 0.602 |
| Anxiolytics | 5 (6.8) | 11 (15.5) | 0.093 |
| Anticonvulsants | 7 (9.5) | 9 (12.7) | 0.537 |
| Blood coagulants | 8 (10.5) | 6 (8.5) | 0.668 |
| Antihypertensive agent | 5 (6.8) | 6 (8.5) | 0.700 |

^*^Categorical variables are represented as number (percentage).

**Table S3. Comparison of baseline mutation rates between the T-705 treated group and the control group.**

|  | **Control group** | **T-705 treated group** | **P value** |
| --- | --- | --- | --- |
| Total mutation rate, mean (CI 95%), % | 0.40 (0.19 to 0.61) | 0.45 (0.26 to 0.63) | 0.706 |
| Transition mutation rate, mean (CI 95%), % | 0.34 (0.16 to 0.53) | 0.36 (0.21 to 0.51) | 0.855 |
| Transversion mutation rate, mean (CI 95%), % | 0.07 (0.03 to 0.11) | 0.08 (0.04 to 0.13) | 0.553 |

**Table S4. The severe fever with thrombocytopenia syndrome questionnaire.**

| Patient Questionnaire | |
| --- | --- |
| Please provide accurate information, and the information will not be disclosed to anyone. | |
| Items | Content |
| Record | _________________________________ |
| A. Demographic Characteristic |  |
| Sex | □Male □Female |
| Age | ______years |
| Occupation | _________________________________ |
| Tel/Mobile | _________________________________ |
| Address | _________________________________ |
| B. Previous history of diseases |  |
| What health/disease did you have in past | _________________________________ |
| Is there any medical treatment going on | _________________________________ |
| If yes, report the kind of medical treatment | _________________________________ |
| Did you have hepatitis | □Yes □No |
| If yes, report the kind of hepatitis | □HAV □HBV □HCV □HDV □HEV |
| Did you have syphilis | □Yes □No |
| Have you been infected with other pathogens | □Rickettsia □Borrelia burgdorferi  □Bebesia microti □No |
| Did you have severe chronic diseases | □Yes □No |
| If yes, report the kind of disease | □HIV □Diabetes □Cancer □Pulmonary tuberculosis □Moderate or severe liver dysfunction □Moderate or severe kidney dysfunction or need dialysis □Hematologic or endocrine diseases need prompt therapy  □Others______________ |
| Did you have severe heart injury | □Yes □No |
| Did you have mental illness and not under control | □Yes □No |
| Did you have history of excessive drinking more than 2 years | □Yes □No |
| C. Medicine history |  |
| Did you have a history of hypersensitivity to an antiviral nucleoside-analog drug targeting a viral RNA polymerase | □Yes □No |
| Did you have a history of drug abuse | □Yes □No |
| Have you been using adrenocorticosteroids (except topical preparation) or immunosuppressive drugs | □Yes □No |
| Did you have contraindications for the use of T-705 (i.e. gout and hyperuricemia) |  |
| D. Exposure history |  |
| Have you been bitten by ticks within two weeks | □Yes □No □Don’t know |
| If yes, report the date of tick bites | _________________________________ |
| Did you have contact with SFTS patients within two weeks | □Yes □No □Don’t know |
| If yes, report the date of contact | _________________________________ |
| If yes, report the relationship with the patient | _________________________________ |
| Did you have field activities within two weeks | □Yes □No □Don’t know |
| Did you have contact with animals within two weeks | □Yes □No □Don’t know |
| If yes, report the kind of animals | □Goat □Cattle □Dog □Pig □Cat □Duck □Chicken  □Others_________________________ |
| E. Current illness |  |
| Presence of fever | □Yes □No |
| If yes, report the date | _________________________________ |
| If yes, report the highest temperature | ___________℃ |
| Other signs or symptoms before admission | _________________________________ |
| Did you have antibiotic treatment before admission | □Yes □No |
| If yes, report the kind of medicine | _________________________________ |
| Did you have antiviral treatment before admission | □Yes □No |
| If yes, report the kind of medicine | _________________________________ |

**Table S5. Data on laboratory test results of the patients with severe fever with thrombocytopenia syndrome.**

| Items | Content |
| --- | --- |
| Record | _________________ |
| Laboratory test date | _________________ |
| A. Hemogram analysis |  |
| White blood cell count | _________­­­×10^9^/L |
| Neutrophil count | _________­­­×10^9^/L |
| Lymphocyte count | _________­­­×10^9^/L |
| Monocyte count | _________­­­×10^9^/L |
| Platelet count | _________­­­­­­×10^9^/L |
| Erythrocyte count | _________­­­×10^12^/L |
| Neutrophil percentage | _________­­­% |
| Lymphocyte percentage | _________­­­% |
| Hemoglobin | _________­­­g/L |
| B. Blood biochemical analysis |  |
| Aspartate aminotransferase | _________U/L |
| Alanine transarninase | _________U/L |
| Total protein | _________­­­g/L |
| Albumin | _________­­­g/L |
| Globulin | _________­­­g/L |
| Total bilirubin | _________μmol/L |
| Alkaline phosphatase | _________U/L |
| Gamma-glutamyl transpeptidase | _________U/L |
| Lactate dehydrogenase | _________U/L |
| Creatine kinase | _________U/L |
| Blood urea nitrogen | _________mmol/L |
| Uric acid | _________μmol/L |
| Creatinine | _________μmol/L |
| C. Blood coagulation analysis |  |
| Prothrombin time | _________S |
| Activated partial thromboplastin time | _________S |
| Thrombin time | _________S |
| Fibrinogen | _________­­­g/L |
| International normalized ratio | _________­­­ |

**Table S6. Data on clinical features of the patients with severe fever with thrombocytopenia syndrome.**

| Items | Content |
| --- | --- |
| Record | _________________ |
| Recording date | _________________ |
| A. Routine examination on admission |  |
| Temperature | _______℃ |
| Pulse | _______times/min |
| Respiratory rates | _______times/min |
| SBP | _______mmHg |
| DBP | _______mmHg |
| B. Common signs/symptoms |  |
| Fever | □Yes □No |
| Chills | □Yes □No |
| Headache | □Yes □No |
| Dizzy | □Yes □No |
| Feeble | □Yes □No |
| Myalgias | □Yes □No |
| Arthralgia | □Yes □No |
| Lmphadenopathy | □Yes □No |
| C. Hemorrhagic signs | □Yes □No |
| Ophthalmorrhagia | □Yes □No |
| Petechial | □Yes □No |
| Ecchymosis | □Yes □No |
| Gingival bleeding | □Yes □No |
| Melena | □Yes □No |
| Hematemesis | □Yes □No |
| Hemoptysis | □Yes □No |
| Epistaxis | □Yes □No |
| Macroscopic hematuria | □Yes □No |
| D. Respiratory symptoms |  |
| Cough | □Yes □No |
| Sputum | □Yes □No |
| Dyspnea | □Yes □No |
| E. Gastrointestinal symptoms |  |
| Anorexia | □Yes □No |
| Nausea | □Yes □No |
| Vomit | □Yes □No |
| Abdominal pain | □Yes □No |
| Diarrhea | □Yes □No |
| F. Neurological symptoms |  |
| Dysphoric | □Yes □No |
| Convulsion | □Yes □No |
| Confusion | □Yes □No |
| Lethargy | □Yes □No |
| Coma | □Yes □No |
| G. Plasma leakage |  |
| Pleural effusion | □Yes □No |
| Pericardial effusion | □Yes □No |
| Pelvic effusion | □Yes □No |
| H. Other infection |  |
| Pulmonary infection | □Yes □No |
| Bronchitis | □Yes □No |

**Table S7. Data on adverse effects related to the administration of T-705 of the patients with severe fever with thrombocytopenia syndrome.**

| Items | Content |
| --- | --- |
| Record | _________________ |
| Recording date | _________________ |
| Adverse effects |  |
| Shock | □Yes □No |
| Skin allergy | □Yes □No |
| Diarrhea | □Yes □No |
| Nausea | □Yes □No |
| Vomit | □Yes □No |
| Abdominal pain | □Yes □No |
| Asthma | □Yes □No |
| Pneumonitis | □Yes □No |
| Jaundice | □Yes □No |
| Toxic epidermal necrolysis | □Yes □No |
| Mucocutaneocular syndrome | □Yes □No |
| Hemorrhagic colitis | □Yes □No |
| Severe liver dysfunction | □Yes □No |
| Acute renal failure | □Yes □No |
| Neurological symptoms | _________________ |
| Elevated level of blood uric acid | □Yes □No |
| Elevated level of triglyceride | □Yes □No |
| Elevated level of aspartate aminotransferase | □Yes □No |
| Elevated level of alanine transarninase | □Yes □No |
| Others | _________________ |

**References**

1 Ministry of Health PRC. Guideline for prevention and treatment of severe fever with thrombocytopenia syndrome (2010 vesion). *Chinese Journal of Clinical Infectious Diseases*. **4**, 193-194 (2011).

2 Liu, W. *et al.* Case-fatality ratio and effectiveness of ribavirin therapy among hospitalized patients in china who had severe fever with thrombocytopenia syndrome. *Clinical infectious diseases*. **57**, 1292-1299, (2013).

3 Yu, X. J. *et al.* Fever with thrombocytopenia associated with a novel bunyavirus in China. *The New England journal of medicine*. **364**, 1523-1532, (2011).

4 Liu, Q. *et al.* Severe fever with thrombocytopenia syndrome, an emerging tick-borne zoonosis. *The Lancet. Infectious diseases*. **14**, 763-772, (2014).

5 Li, Y. *et al.* Epidemiological analysis on severe fever with thrombocytopenia syndrome under the national surveillance data from 2011 to 2014, China. *Chinese Journal of Epidemiology*. **36**, 598-602, (2015).

6 Kim, K. H. *et al.* Severe fever with thrombocytopenia syndrome, South Korea, 2012. *Emerging infectious diseases*. **19**, 1892-1894, (2013).

7 Takahashi, T. *et al.* The first identification and retrospective study of Severe Fever with Thrombocytopenia Syndrome in Japan. *The Journal of infectious diseases*. **209**, 816-827, (2014).

8 Luo, L. M. *et al.* Haemaphysalis longicornis Ticks as Reservoir and Vector of Severe Fever with Thrombocytopenia Syndrome Virus in China. *Emerging infectious diseases*. **21**, 1770-1776, (2015).

9 Jung, I. Y. *et al.* Nosocomial person-to-person transmission of severe fever with thrombocytopenia syndrome. *Clinical microbiology and infection*. **25**, 633 e631-633 e634, (2019).

10 Tang, X. *et al.* Human-to-human transmission of severe fever with thrombocytopenia syndrome bunyavirus through contact with infectious blood. *The Journal of infectious diseases*. **207**, 736-739, (2013).

11 Oh, W. S. *et al.* Plasma exchange and ribavirin for rapidly progressive severe fever with thrombocytopenia syndrome. *International journal of infectious diseases*. **18**, 84-86, (2014).

12 Li, H. *et al.* Epidemiological and clinical features of laboratory-diagnosed severe fever with thrombocytopenia syndrome in China, 2011-17: a prospective observational study. *The Lancet. Infectious diseases*, **18,** 1127-1137, (2018).

13 Vanderlinden, E. *et al.* Distinct Effects of T-705 (Favipiravir) and Ribavirin on Influenza Virus Replication and Viral RNA Synthesis. *Antimicrobial agents and chemotherapy*. **60**, 6679-6691, (2016).

14 Mendenhall, M. *et al.* T-705 (favipiravir) inhibition of arenavirus replication in cell culture. *Antimicrobial agents and chemotherapy*. **55**, 782-787, (2011).

15 Morrey, J. D. *et al.* Efficacy of orally administered T-705 pyrazine analog on lethal West Nile virus infection in rodents. *Antiviral Res*. **80**, 377-379, (2008).

16 Gowen, B. B. *et al.* In vitro and in vivo activities of T-705 against arenavirus and bunyavirus infections. *Antimicrobial agents and chemotherapy*. **51**, 3168-3176, (2007).

17 Guedj, J. *et al.* Antiviral efficacy of favipiravir against Ebola virus: A translational study in cynomolgus macaques. *PLoS medicine*. **15**, e1002535, (2018).

18 Scharton, D. *et al.* Favipiravir (T-705) protects against peracute Rift Valley fever virus infection and reduces delayed-onset neurologic disease observed with ribavirin treatment. *Antiviral Res*. **104**, 84-92, (2014).

19 Tani, H. *et al.* Efficacy of T-705 (Favipiravir) in the Treatment of Infections with Lethal Severe Fever with Thrombocytopenia Syndrome Virus. *mSphere*. **1**, e00061-15, (2016).

20 Gowen, B. B. *et al.* Modeling Severe Fever with Thrombocytopenia Syndrome Virus Infection in Golden Syrian Hamsters: Importance of STAT2 in Preventing Disease and Effective Treatment with Favipiravir. *J Virol*. **91**, e01942-16, (2017).

21 MediVector, Inc. A phase 3, randomized, double-blind, placebo-controlled, multicenter study evaluating the efficacy and safety of favipiravir in adult subjects with uncomplicated influenza. https://[www.clinicaltrials.gov./ct2/show/NCT02008344?term=favipiravir&rank=3](http://www.clinicaltrials.gov./ct2/show/NCT02008344?term=favipiravir&rank=3). Jan 30 (2016). .

22 Petrosillo, N. *et al.* Ebola virus disease complicated with viral interstitial pneumonia: a case report. *BMC infectious diseases*. **15**, 432, (2015).

23 Schibler, M. *et al.* Clinical features and viral kinetics in a rapidly cured patient with Ebola virus disease: a case report. *The Lancet. Infectious diseases*. **15**, 1034-1040, (2015).

24 Chinello, P. *et al.* QTc interval prolongation during favipiravir therapy in an Ebolavirus-infected patient. *PLoS Negl Trop Dis*. **11**, e0006034, (2017).

25 Raabe, V. N. *et al.* Favipiravir and Ribavirin Treatment of Epidemiologically Linked Cases of Lassa Fever. *Clinical infectious diseases*. **65**, 855-859, (2017).

26 Bai, C. Q. *et al.* Clinical and Virological Characteristics of Ebola Virus Disease Patients Treated With Favipiravir (T-705)-Sierra Leone, 2014. *Clinical infectious diseases*. **63**, 1288-1294, (2016).

27 Sissoko, D. *et al.* Experimental Treatment with Favipiravir for Ebola Virus Disease (the JIKI Trial): A Historically Controlled, Single-Arm Proof-of-Concept Trial in Guinea. *PLoS medicine*. **13**, e1001967, (2016).
